# Posterior Communicating Artery Aneurysm Microsurgery: PComA-CORE, an Anatomy-Informed Explainable AI Framework for Complexity, Neurovascular Risk, Oculomotor Recovery and Functional Outcome

**DOI:** 10.3390/medsci14050528

**Published:** 2026-08-28

**Authors:** Matei Șerban, Corneliu Toader, Alexandru Vlad Ciurea, Leon Dănăilă, Răzvan-Adrian Covache-Busuioc

**Affiliations:** 1 Department of Neurosurgery, “Carol Davila” University of Medicine and Pharmacy, 050474 Bucharest, Romania; mateiserban@innbn.com (M.Ș.);; 2 Department of Vascular Neurosurgery, National Institute of Neurology and Neurovascular Diseases, 077160 Bucharest, Romania; 3 Puls Med Association, 051885 Bucharest, Romania; 4 Medical Section, Romanian Academy, 010071 Bucharest, Romania

**Keywords:** posterior communicating artery aneurysm, intracranial aneurysm, microsurgical clipping, artificial intelligence, machine learning, explainable artificial intelligence, risk stratification, oculomotor nerve palsy

## Abstract

The posterior communicating artery (PComA) aneurysm is a challenging microsurgical problem due to multiple factors. These include the technical complexity of the surgery itself, potential injury to blood vessels during surgery, recovery of cranial nerve function, and overall neurological outcome after the operation. These are all related to the area where the PComA aneurysm is located, but they have fundamentally different biological determinants. Prior methods of describing aneurysms do not adequately describe how the relationships of the internal carotid artery (ICA) and PComA/P1 configuration influence the proximity of the aneurysm to other important structures such as the perforating arteries, the anterior choroidal artery (AChA), cranial nerve III (CN III), and the surgical corridor. We created PComA-CORE, an artificial intelligence-based framework designed to evaluate whether the elements of experienced microsurgeons’ thought processes can be measured individually while still maintaining temporally valid predictions, human interpretability, and explicit estimates of predictive uncertainty. **Methods:** Using a highly detailed database of clinical, radiographic, anatomical, intraoperative, and longitudinal data from 687 adult patients who underwent microsurgical clipping of PComA aneurysms over the period 1997–2026, we applied PComA-CORE to predict separately: Microsurgical Complexity (C); Oculomotor Recovery (O); Neurovascular Preservation Risk (R); and Expected 90-Day Functional Outcome (E). The models used cases from 1997–2020 (*n* = 564) for development and cases from 2021–2026 (*n* = 123) for temporal evaluation. Several architectures, including penalized regression, machine-learning techniques, interpretable machine learning, and ensembles, were compared using nested cross-validation, discrimination metrics, calibration metrics, decision-curve analysis, explainability measures, uncertainty-aware prediction, inter-observer reproducibility, and model-to-score distillation. **Results:** Four discrete predictive architectures were identified by PComA-CORE. PComA-C was found to be highly dependent upon anatomy because the specific geometric characteristics of individual vascular segments and the presence or incorporation of branches around the aneurysm strongly influenced temporal predictions. PComA-R was found to behave as a distributed susceptibility phenotype based on neurovascular attributes rather than a deterministic injury model and achieved a temporal AUC of 0.703. Among patients with preoperative CN III palsy, PComA-O identified that recovery primarily depended upon the time course of neurological dysfunction and structural deformation of the affected nerve. Temporal validation was not feasible given the small number of recent non-recovery events. Conversely, PComA-E showed that global functional outcome continued to depend predominantly upon clinical neurological severity, with a temporally evaluated penalized model achieving an AUC of 0.878. Uncertainty-aware predictions indicated that some cases would benefit from greater caution in interpretation. High-resolution anatomical phenotypes demonstrated good inter-observer reproducibility. Score distillation demonstrated that simplification preserved predictive information, but did so differently depending on the endpoint. **Conclusions:** The problem of predicting the consequences of clipping a PComA aneurysm is multidimensional and does not exist as a single “risk” prediction problem. Technical complexity, neurovascular vulnerability, neural recovery, and global disability each exist within distinct predictive spaces and require different levels of anatomical detail and/or computational complexity. PComA-CORE establishes a human-supervised framework to transform expert microsurgical thought processes into explicit, reproducible, uncertainty-aware, and clinically interpretable representations. While prospective multicenter validation will be needed prior to clinical use, it has the potential to establish a basis for explainable AI, precision cerebrovascular neurosurgery, anatomy-informed risk stratification, and clinically interpretable decision-support systems in complex aneurysm surgery.

## 1. Introduction

High-resolution imaging of intracranial vascular anatomy provides unique detail regarding the interactions between arteries and nerves. These details can directly affect the technical difficulty of aneurysm repair and patient outcomes. The microsurgical challenge presented by a posterior communicating artery (PComA) aneurysm is related to the close proximity of the aneurysm to several critical structures, including the internal carotid artery (ICA), P1 segment and posterior cerebral artery (PCA), anterior choroidal artery (AChA), perforators, CN III, and the surgical corridors [1]. For example, a small aneurysm located near the junction of the ICA and PComA may present very different challenges from a larger aneurysm that incorporates multiple branches or perforators. Further, two aneurysms of identical size located at similar anatomical sites may present different challenges based upon their respective relationships to CN III and their resultant effects on oculomotor function. These variations cannot be captured fully by simple classification systems based on aneurysm size, location, or angiographic occlusion status [2].

The primary goal of microsurgical clipping of PComA aneurysms is to preserve neurovascular function in conjunction with successful exclusion of the aneurysm. Successful exclusion must be achieved without compromising PComA/PCA blood flow, while maintaining patency of the AChA and supplying perforators and avoiding permanent injury to CN III [3]. These goals typically require careful dissection within limited working spaces, management of calcification and thrombus, preservation of incorporated branches, consideration of variable fetal circulation patterns, and management of unusual dome projections. Preservation of macroscopic blood flow through the above-mentioned vessels does not ensure the absence of tissue-level ischemic injury [4]. Moreover, anatomical contact between a PComA aneurysm and CN III does not predictably correlate with the degree or persistence of oculomotor dysfunction. Therefore, microsurgical complexity, preservation of neurovascular function, recovery of CN III function, and global functional outcome constitute distinct aspects of an integrated approach to treating a PComA aneurysm [5].

It is challenging to retain these distinctions in current aneurysm databases. Standardized registries efficiently document what happened—i.e., rupture, treatment method, occlusion status, infarction, disability level, need for retreatment, or death—but they do not as commonly record why an operation was technically complicated, which anatomical configurations created vulnerability, when this vulnerability was recognized, or how intraoperative verification and corrective actions related to subsequent tissue injury [6]. Anatomically significant variability in the clinically relevant risks associated with PComA aneurysms likely arises from concurrent involvement of several anatomical elements rather than being confined to a single measurable feature [7]. For instance, the clinical relevance of fetal PCA configuration depends on the adequacy of the P1 segment and collateral circulation; branch incorporation is influenced by both neck geometry and reconstructive feasibility; hidden perforators may alter otherwise favorable aneurysm morphology; and CN III recovery may depend on both the duration of preoperative oculomotor dysfunction and the degree of structural deformation of CN III. As a result, we considered it important to develop our predictive models using data that maintained both high anatomical resolution and temporal eligibility [8].

Machine learning and artificial intelligence technologies allow investigators to assess nonlinear associations between anatomically correlated variables and higher-order interactions that traditional statistical models may not fully capture. However, increased computational complexity does not necessarily equate to increased clinical utility. A model can have excellent discriminatory ability yet still be difficult to use clinically if it is poorly calibrated, unstable over time, relies upon data that are not available at the intended point of clinical decision-making, or fails to translate its results into recognizable anatomical relationships [9]. In contrast, more parsimonious penalized or interpretable models may be preferred when increasingly complex models provide little additional reproducible information. We therefore did not seek merely to identify the best-performing algorithm. Rather, we sought to determine whether high-resolution PComA anatomy contained reproducible information beyond that contained in standard clinical and morphological descriptions [10]. We also sought to determine whether this information remained stable over time, where and how predictive uncertainty arose, and how much of the computational signal could ultimately be condensed into clinically usable instruments.

To answer these questions, we retrospectively analyzed 687 microsurgically treated PComA aneurysms over a 30-calendar-year period from 1997 through 2026, using a longitudinal neurovascular database containing 779 variables spanning clinical, anatomical, operative, postoperative, and longitudinal domains. Rather than evaluating PComA aneurysm treatment through a single composite endpoint, we created the PComA-CORE framework to examine four mechanistically distinct but clinically interrelated issues surrounding microsurgical treatment of PComA aneurysms: C—PComA-C, estimating pre-incision microsurgical complexity; O—PComA-O, assessing the probability and longitudinal trajectory of recovery from preoperative CN III palsy; R—PComA-R, identifying susceptibility to procedure-related ischemia within the PComA–P1–PCA–AChA–perforator network; and E—PComA-E, estimating expected 90-day functional outcome while preserving the complete ordinal mRS spectrum. Predictor eligibility for each component was specifically constrained by time so that information acquired during or after surgery could not retrospectively influence predictions intended to be available before incision.

We further wished to explore whether the aforementioned four questions utilized a common predictive architecture or alternatively required substantially different representations of clinical and anatomical information. Thus, we employed conventional and penalized regression techniques, nonlinear machine-learning algorithms, interpretable AI methods, survival modeling, temporal validation, anatomical domain-specific analyses, uncertainty-aware prediction, and sparse integer optimization as complementary analytical and benchmarking layers rather than merely as examples of increasing computational sophistication. Our intention was not to create an automated surgical recommendation system or to supplant the clinician’s judgement. Rather, we aimed to establish whether microsurgically relevant information encoded within high-resolution PComA anatomy could be formally expressed as reproducible estimates of complexity, oculomotor recovery, neurovascular risk, and expected functional outcome. Furthermore, we sought to determine whether clinically meaningful information could be preserved as these predictions were reduced from high-dimensional computational models into more practical and portable PComA-CORE instruments suitable for subsequent independent multicenter and prospective validation.

## 2. Materials and Methods

### 2.1. Study Design and Dataset Description

This is a retrospective, single-center model-development study to determine if high-dimensional anatomical and microsurgical information obtained from a longitudinal posterior communicating artery (PComA) aneurysm registry can be translated into clinically interpretable and reproducible predictive representations. Due to the compact nature of the neurovascular interface within the PComA region, the aneurysm’s neck and dome, PComA origin, P1 segment and posterior cerebral circulation, anterior choroidal artery (AChA), perforating arteries, internal carotid artery (ICA), oculomotor nerve (CN III), and microsurgical corridors are usually located in close proximity to one another (i.e., only a few millimeters apart). Since conventional descriptions of aneurysms (e.g., aneurysm size, rupture status, angiographic occlusion, etc.) do not sufficiently describe how difficult it may be to perform surgery on the aneurysm and what level of risk the patient will incur during surgery, aneurysms that are of similar size may have large differences based on their fetal posterior-circulation dependency, P1 hypoplasia or absence, AChA or perforator involvement, retro-dome perforators, CN III compression or groove formation, and the limiting influence of the carotid-oculomotor corridor. Thus, PComA-CORE was created using four anatomically related yet biologically distinct predictive tasks: PComA-C, estimating the complexity of microsurgical procedures prior to making an incision; PComA-O, determining the likelihood and timeline of recovery from preoperative CN III palsy; PComA-R, measuring a patient’s susceptibility to procedure-induced neurovascular ischemia; and PComA-E, evaluating a patient’s 90-day functional status. This distinction was made to preserve the separation between technical difficulty, local cranial nerve recovery, vascular injury, and overall disability and not to combine them all into a single composite risk index.

The analytical framework employed traditional and penalized regression methods, non-linear machine-learning methods, interpretable AI algorithms, current table-based benchmarking methods, survival learning to estimate CN III recovery timelines, and sparse integer optimization to distill the models. Further complexity was not seen as desirable solely because it added complexity; further complexity was only warranted when it contributed to stable gains in discrimination, calibration, interpretability, or clinically interpretable information. Model outputs did not affect treatment decisions, operative planning, intraoperative decision-making, postoperative management, or follow-up.

Therefore, this study should be viewed as a model-development study with internal resampling and same-center temporal assessment rather than external validation. Thus, independent multicenter validation is required before clinical application. Reporting was accomplished according to the TRIPOD + AI reporting standards and incorporated PROBAST + AI criteria for participant inclusion (demographics), defining predictors (anatomy), ensuring outcome integrity (reliability), minimizing overfitting (cross-validation), validating results (bootstrapping), calibrating models (Brier scoring), and applicability (transferability) [11].

Patient Population, Definitions of Anatomy, Inclusion Criteria, and Exclusions

The registry included 687 adults operated on for PComA aneurysms between 1997 and 2026 at the National Institute of Neurology and Neurovascular Diseases in Bucharest, Romania. To guarantee anatomical homogeneity of cases evaluated within the registry, PComA aneurysms were defined as lesions arising from the ICA-PComA junction or directly from the PComA. Cases were grouped by anatomical location into three groups: ICA-PComA junction; true proximal PComA; and true mid/distal PComA aneurysms. Cases derived from the AChA, ICA terminus, cavernous ICA, or PCA without direct PComA origin were excluded from evaluation.

Inclusion criteria included age ≥ 18 years; confirmed vascular imaging documenting a PComA lesion; surgical repair of the aneurysm; sufficient predictor information available throughout the study period; and documented data relating to the endpoint(s) being studied. Eligibility for analyses concerning previously documented outcomes continued for patients who met inclusion criteria regardless of subsequent missing follow-up data. Only patients meeting the inclusion criteria listed above were eligible for enrollment in PComA-O if documentation demonstrated preoperative CN III palsy resulting from the aneurysm. Analysis of variables associated with SAH was confined to ruptured aneurysms or rupture-gated models.

In total, 332 (48.3%) aneurysms in the cohort were ruptured, while 355 (51.7%) were unruptured. The median age of subjects was 59.1 years (IQR: 50.4–68.4), and females comprised 531 (77.3%) of the subjects participating in the study. Subjects with aneurysms originating at the ICA-PComA junction comprised 95.1% of subjects (*n* = 653); true proximal PComA aneurysms comprised 3.6% of subjects (*n* = 25); and true mid/distal PComA aneurysms comprised 1.3% of subjects (*n* = 9). Adult-type posterior circulation was noted in 77.6% of subjects (*n* = 533); partial fetal in 12.8% of subjects (*n* = 88); and complete fetal in 9.6% of subjects (*n* = 66). Documentation of preoperative CN III palsy was observed in 25.5% of subjects (*n* = 175); partial palsies were documented in 56.6% of subjects (*n* = 99); complete palsies were documented in 43.4% of subjects (*n* = 76). Median maximum aneurysmal diameter was 6.2 mm (IQR: 4.5–8.9); median neck width was 2.8 mm (IQR: 1.7–4.4). Median Hunt–Hess grade was 2; median WFNS grade was 2; median GCS score was 13; and median modified Fisher grade was 3 among patients with ruptured aneurysms (Table 1).

The present study received approval from the Institutional Ethics Committee (Research Project No. 4623, approved on 25 May 2026), and all data used for statistical analysis had been anonymized before analysis. All data management followed the rules of the institution concerning the confidentiality of individually identifiable health information, as well as the corresponding Romanian and EU legislation regarding the protection of personal and health-related data. Thus, we removed from predictive modeling all variables that could potentially identify patients or operators, including de-identification metadata, institutional linkage keys, clinician identifiers, administrative identifiers, and free-text fields capable of functioning as identifiers for individual patients or operators.

Registry Development, Longitudinal Harmonization & Data Collection

The registry is not a new registry designed specifically for this study’s artificial intelligence (AI) analysis. Rather, it is a registry that has been in conceptual development for approximately thirty years within the institutional academic neurosurgery program led by senior neurosurgeons. The conceptual basis for developing a repository of clinically relevant and microsurgically relevant neurovascular information longitudinally commenced well before advances in vascular imaging technology, microsurgical techniques, intraoperative verification techniques, and postoperative assessment techniques. Hence, as technological developments evolved, so too did the registry, while retaining consistent clinical and anatomical architectures pertinent to neurological deficits due to vascular anomalies, vascular anatomy relevant to surgical intervention, postsurgical complications, recovery, and long-term durability.

Prior registry data were synchronized into an analytical framework consisting of 779 analytically defined variables employing a version-controlled data dictionary containing clearly defined clinical constructs, anatomical constructs, data type(s), unit(s) if applicable, source domain(s), temporal layer(s), missing-value indicator(s), predictor eligibility indicator(s), and derivation indicator(s). All variables collected across different time frames were uniformly defined only if there existed sufficient similarities in their clinical constructs and interpretation of measurement. Variables lacking definable units or definitive interpretations, or with insufficient information to reasonably translate them into comparable units, were either placed into quarantine or removed rather than being force-normalized.

Therefore, the variables are reflective of all available longitudinal data contained within the registry as opposed to forming a hypothetical cohort wherein each subject had a comprehensive array of measurements taken at each time interval. Although many patients had completely documented data throughout all levels of the registry architecture, other patients’ individual variables were restricted based upon clinical necessity, quality of original source documentation, imaging modalities utilized during diagnosis, technology available during intraoperative interventions, treatment period, and/or duration of follow-up. Furthermore, in most instances, lack of documentation was differentiated from documentation of a negative finding and variables dependent upon technology unavailable until later periods were not assumed to be indicative of biological absence. Consequently, missing values were individually addressed for each variable as opposed to assuming they represented uniform limitations based upon historical eras.

Demographic variables, along with pre-existing conditions, extent of premorbid functioning ability, clinical comorbidity status, neurological examination findings, and documentation regarding whether an aneurysmal rupture had occurred, were considered as part of the clinical variables in addition to GCS scores; Hunt–Hess grades; WFNS grading system scores; Fisher grades; modified Fisher grades; Hijdra blood-burden scores; IVH; ICH; hydrocephalus; cerebral edema; rebleeding; and rupture-to-treatment delay times.

Morphologic variables describing aspects of aneurysm and vessel morphology consisted of maximum diameter and neck width; dome-to-neck ratio; aspect ratio; size ratios; volume; surface area; presence/absence of lobularity; presence/absence of irregularity; presence/absence of daughter sac(s); presence/absence of bleb(s); presence/absence of thrombus; presence/absence of calcifications; presence/absence of atherosclerosis in the parent artery; primary projection characteristics; secondary projection characteristics; plane orientation relative to the neck plane and inflow angle; internal carotid artery caliber and tortuosity; relationship to the clinoidal process and optic nerve; opticocarotid corridor; and carotid–oculomotor corridor. Variables characterizing PComA-P1-PCA consisted of adult-type configuration; partial fetal-type configuration; complete fetal-type configuration; diameter of PComA and P1 arteries; presence/absence of P1 hypoplasia or aplasia; relationships between PComA and P1 arteries; presence/absence of adequate collaterals; presence/absence of the contralateral P1 artery; and dependency on posterior circulation. Variables characterizing AChA consisted of diameter; visibility; distance from the aneurysm neck; branching patterns; incorporation into the neck/sac; and adherence to nearby vessels.

Perforator topology was represented individually through count; origin; incorporation into the neck or sac of the aneurysm; adherence to surrounding tissues; stretching by the aneurysm; concealment behind the aneurysm dome; origin from the PComA/P1/ICA; premammillary artery identity and origin; tuberoinfundibular anatomy; complexity of thalamoperforator relationships; presence/absence of hypothalamic perforator risk; and confidence in creating a topographical map of these branches.

Finally, CN III was described both anatomically and longitudinally as a phenotypic expression. Prior to surgery, CN III was characterized using the following preoperative variables: presence/absence/partial/complete palsy; duration since symptom onset; timing of symptom onset; presence/absence/duration/severity of pupillary dysfunction; degree/severity/location of ptosis; degree/severity/localization of impairment of horizontal eye movement/adduction/vertical eye movement; diplopia; pain; and anatomical deficit severity. Anatomical features documented in relation to CN III included contact with the aneurysm; length of contact with the aneurysm; displacement of CN III; compression grade; grooving; stretching; adherence of the sac to CN III; degree of compression caused by thrombus; cisternal-segment involvement; and distance between the aneurysm and CN III. Postoperative assessments documented first improvement in CN III function, recovery to normal/functionally intact status, and residual ptosis/diplopia/pupillary dysfunction/ophthalmoplegia at each time point (30 days/90 days/180 days/365 days/730 days). Since the predictive framework was designed to illustrate mechanisms involved in surgical intervention for PComA aneurysms rather than administrative outcomes alone, the registry was organized around anatomically and clinically relevant information domains encompassing vascular abnormalities, operative anatomy, postsurgical complications, recovery and long-term durability, as summarized in Table 2.

Preventing Information Leakage Using Temporal Gates

Information leakage was prevented primarily by gating processes. It was determined when a variable would first be measurable (i.e., available). All features necessary to make predictions prior to incision were constrained to utilize only those measures available prior to that time. Operative and postoperative variables were permitted to represent future states of either endpoints or mechanisms of injury but were prohibited from providing evidence about past events occurring prior to their collection.

The temporal structure thus ensured that no postoperative data could contribute evidence toward a preoperative prediction model; that no composite scores reflecting good outcomes could be used to forecast individual component mRS values; that no composite scores forecasting branch preservation could be used to forecast vascular outcomes pertaining to their respective branches; that postoperative CN III status could not serve as evidence for preoperative PComA-O; and that postoperative stroke or death could not be used to forecast either event (Table 3).

### 2.2. Data Preprocessing and Feature Engineering

The steps for data preprocessing and feature engineering included examining the presence of duplicate data, checking whether the data from different domains were internally consistent, analyzing the percentage of missing data, determining whether each variable had values within acceptable numeric ranges and reasonable units, and utilizing computer tools to identify duplicated variables or variables very similar to one another. Additionally, it was examined whether any deterministic pairs of predictors and outcomes existed. All features with uncertain, unknown, or improbable unit designations, unreasonable numeric ranges, or inadequate sample sizes to produce a valid numeric assessment were designated as “quarantined” and thus excluded from retroactive rescaling.

Non-transportable identifiers (e.g., clinician IDs), administrative linkage metadata, and free-text fields capable of identifying individual patients or operators were removed. Missingness was characterized before model-specific preprocessing, with structural non-applicability distinguished from conventional missingness. Variables were considered structurally non-applicable when the corresponding clinical condition or analytical context was absent, and outcomes were never imputed. Table 4 provides the corresponding domain-level completeness, principal sources of unavailable information, and analytical handling of missing data, whereas Table 5 summarizes data availability for the principal clinical, anatomical, and outcome variables.

In the training phases, missing values in the predictor matrix were handled differently based on the specific predictor(s) in the relevant training partitions. Predictor features were treated as either continuous or categorical. Linear interpolation, where possible, and k-nearest-neighbor methods for continuous predictors, and conditional random fields for categorical predictors, were used during training. Missingness indicators were added when absence of measurements themselves indicated treatment era, imaging availability, or documentation practices. All imputation procedures were strictly limited to the training partitions so that validation or temporal-evaluation data could not inform preprocessing.

Wherever possible, continuous features remained on their original scales; otherwise, restricted cubic splines were utilized to explore potential nonlinear effects. Continuous predictors were not arbitrarily categorized or dichotomized unless for descriptive purposes or for utilization in sparse bedside scores. Depending on the algorithm, encoding and normalization of categorical variables were performed. CatBoost supported categorical variables directly; scaling was applied only where processing depended on the magnitude of the predictors. All preprocessing parameters, including those associated with imputation, scaling, and transformation, were derived exclusively from training data.

Candidate predictors were successively narrowed down based on clinical-domain applicability, temporal eligibility (clinical plausibility), and necessary conditions for the algorithm. Candidate predictors had to meet requirements of clinical relevance, practical measurability, no direct outcome leakage, and external reproducibility. Variables that were highly correlated or anatomically redundant were analyzed using correlation matrices, hierarchical clustering methods, variance inflation factor diagnostics, and domain-specific redundancy analyses. Candidate predictors were not chosen solely due to statistically significant univariate *p*-value comparisons.

Two nested predictor spaces were defined. The Universal PComA feature space comprised clinical and vascular imaging-based information that can generally be collected in patients. This served as a basis for developing the bedside scoring system. The Extended Anatomical feature space extended beyond typical clinical and vascular imaging-based information by incorporating additional high-resolution morphometric, geometric, and advanced imaging descriptors to determine whether detailed PComA anatomy provides additional predictive value compared with clinical and vascular imaging-based information generally obtainable in patients.

### 2.3. Development of the PComA-CORE Framework

The four PComA-CORE framework components (microsurgical complexity, oculomotor recovery, neurovascular preservation, and expected functional outcome) share a common methodological foundation. However, due to the differing biological mechanisms underlying the various components—eligible populations (i.e., patients); different prediction times (e.g., immediate perioperative vs. intermediate and late recovery); varying endpoints; and different predictive domains—the four components were developed independently.

#### 2.3.1. PComA-C: Microsurgical Complexity Score

The microsurgical complexity score quantifies the level of difficulty for the surgeon to perform the surgical procedure prior to making the incision. The principal outcome measure for this analysis is a registry-defined high-complexity operative phenotype that was observed in 122 of 687 cases (17.8%); as opposed to postoperative complications or longer-term functional outcomes, the definition is based solely on the degree of operative effort needed. Examples of items included in this assessment are: difficulty in exposing the aneurysm; requirement for additional reconstructive procedures; prolonged temporary occlusion; need for multiple revisions/repositionings of clips; need for flow-rescue maneuvers; and intraoperative rupture requiring increased vascular control. At least two of the complexity indicators must be met for classification as a high-complexity operative phenotype. In addition, ≥3 attempts at clip repositioning or the use of any flow-rescue maneuver alone will suffice for meeting this criterion.

Examples of preoperative predictors that can be employed as correlates of aneurysm characteristics include aneurysm size and neck geometry; projection and shape; presence and/or extent of thrombosis and calcification; ICA relationship to the clinoid process and optic/CN III structures; carotid–oculomotor and opticocarotid corridors; fetal PCA configuration; P1 hypoplasia or absence; relationship between the PComA/AChA and the aneurysm neck/sac; perforator topography including number and/or location of retro-dome perforators; and rupture status.

Variables that relate to surgery—including the actual number of clips applied during surgery; total time spent applying temporary clipping; need for greater access; use of flow-rescue maneuvers; rupture occurring during surgery; and clip repositioning—can only be used to establish identification of the operative phenotype and therefore cannot be used as predictors prior to incision. This differentiation preserves the temporal distinction between predictor values and the technical burden that they are intended to predict.

#### 2.3.2. PComA-O: Oculomotor Recovery Score

Only 175 patients presenting with preoperative CN III palsy resulting from their aneurysms were eligible for prediction of oculomotor recovery. Complete recovery of CN III function occurred in 112 patients at 90 days post-microsurgery and in 155 patients at one year. The primary outcome measure for this analysis is the time from completion of microsurgery until documentation of complete recovery. Patients whose recovery was not documented were censored at their date of last known follow-up; death prior to recovery was treated as a competing event in sensitivity analyses.

Eligible predictors included: whether there was partial versus complete CN III palsy prior to surgery; how long before surgery symptoms began; whether pupils were affected; presence of ptosis; inability to adduct, elevate, or depress the eye; diplopia; degree of CN III deficit; aneurysm size/projection; rupture status; proximity between the aneurysm and CN III; degree of contact/compression between the aneurysm and CN III; grade of grooving/stretching of CN III by the aneurysm sac; degree of adherence between the aneurysm sac and CN III; whether thrombosis resulted in compression of CN III; cisternal-segment involvement; and degree of PComA configuration. All intraoperative manipulations/maneuvers involving CN III—including decompression and post-clip tension release—can only be evaluated retrospectively as mechanistically derived updates. Secondary outcomes for recovery included complete recovery at 90 days, 12 months, and 24 months, as well as persistent ptosis, diplopia, or pupillary dysfunction at 12 months.

Because only 30 patients who underwent microsurgery between 2021 and 2026 had preoperative CN III palsy, and only two failed to achieve complete recovery within the first year, PComA-O was designated as exploratory and intended only for derivation purposes. As such, no conclusions were made concerning external validity. Rather, it underwent a same-center temporal stress test utilizing data from a subsequent cohort.

#### 2.3.3. PComA-R: Neurovascular Preservation Risk Score

PComA-R assesses the likelihood of damage to specific areas within the neurovascular network, i.e., the PComA–P1–PCA–AChA–perforator network. The principal outcome measure is procedure-related ischemic injury, which was identified in 87 of 687 patients (12.7%). Failure to preserve critical branches occurred in 28 patients and served as a secondary mechanistic outcome measure. Ischemic injury and branch-preservation failure were also evaluated in terms of anatomically concordant ischemia/critical branch compromise, symptomatic infarcts affecting the AChA and PCA territories, premammillary/infrathalamic infarcts, thalamic/internal capsular infarcts, and a composite anatomically concordant vascular endpoint.

Examples of eligible predictors include: posterior-circulation configuration; PComA/P1 diameter/diameter ratio/dependency; degree of P1 hypoplasia or absence; adequacy of the contralateral P1; degree of incorporation/stretching/adherence between the PComA and adjacent structures; AChA diameter/neck distance; degree of incorporation of the AChA into surrounding structures; number/origin/incorporation of perforators into surrounding structures; presence/number/location of retro-dome perforators; premammillary/hypothalamic/thalamoperforator anatomy; aneurysm morphology/projection/geometry/neck; presence/degree of calcification; ICA geometry; and rupture status.

As noted previously, postoperative patency, DWI infarct, and intraoperative rescue maneuvers represent mechanistically derived outcomes or states but are not predictors of their occurrence. Because fewer ischemic events were reported relative to the number of potential anatomical predictors, emphasis was placed on regularization methods, nested cross-validation, bootstrapping/resampling, selection of eligible predictors, and consideration of temporal effects during model development. Nonetheless, residual overfitting remains possible and should be acknowledged when evaluating the results.

#### 2.3.4. PComA-E: Expected Functional Outcome Score

PComA-E predicts functional outcomes 90 days post-surgery. We therefore retained the entire ordinal scale (0–6) of modified Rankin Scale (mRS) scores as our primary outcome measure. For the 635 patients with 90-day mRS scores available, 468 (73.7%) achieved mRS scores ≤ 2, while 167 (26.3%) had mRS scores > 2. The latter dichotomy was retained as a secondary outcome so we could evaluate our findings against existing neurovascular studies and generate bedside-based scoring systems. To provide a concise overview of endpoint availability, event support, and analytical eligibility across the four PComA-CORE components, Table 6 summarizes the endpoint-specific populations and outcome distributions used for PComA-C, PComA-R, PComA-O, and PComA-E.

Hierarchical modeling allowed us to create our final model for predicting mRS 90 days post-surgery. The first model consisted exclusively of demographic and clinical variables including age, premorbid function, rupture status, admission neurological severity, SAH burden, and hydrocephalus if present. The second model included detailed morphometric features describing PComA anatomy including fetal configuration, P1 dependency, AChA/perforator anatomy, CN III phenotype, and spatial relationships among neurovascular elements. Hierarchical models permitted us to determine the incremental contributions provided by detailed PComA anatomy beyond baseline neurological severity (Table 7).

### 2.4. Data Splitting and Internal/Temporal Validation Strategy

Rather than relying on a random 80/20 split, we considered that such an approach might be insufficient for a database reflecting substantial changes in medical practice related to cerebrovascular disease. Thus, we assigned all case material collected between 1997 and 2020 to the development group (*n* = 564) and cases collected between 2021 and 2026 to the temporally separated same-center evaluation group (*n* = 123). The latter cohort included 22 high-complexity cases, 18 procedure-related ischemic complications, and 117 patients for whom 90-day mRS values were available, including 22 who had poor outcomes.

No data points from the temporally separate evaluation group were employed in selecting features to include in the model, developing rules for imputing missing data, optimizing model parameters, deriving scoring models, determining optimal thresholds for classifying patients into risk categories, constructing ensembles of classifiers, or calibrating classifiers prior to evaluating their performance. This temporally separated same-center design assessed how well our machine-learning classifiers performed in terms of within-center temporal portability but did not provide external validation, since it was based on the same hospital and was therefore likely influenced by similar levels of surgical expertise, measurement practices, and documentation habits.

Nested cross-validation was used to generate strictly out-of-fold estimates of performance in the development group. In turn, outer resampling was used to estimate performance metrics, and inner resampling was used for performing imputation, scaling, feature selection and filtering, interaction selection, parameter optimization, model selection, and calibration. Parameters associated with nonlinear models were optimized using constrained Bayesian optimization within predefined search regions.

We used bootstrap resampling with up to 2000 iterations per endpoint when sufficient events were available to examine optimism, coefficient and feature-selection stability, calibration uncertainty, variability of feature importance, and uncertainty in clinical net benefit. We also compared observed discrimination against distributions derived after disrupting associations between predictors and outcome variables through permutation testing.

The “era” variable was further analyzed as a means of assessing how robustly our models transported across different treatment eras through the use of four strata: 1997–2007, 2008–2014, 2015–2020, and 2021–2026. These eras corresponded to progressively changing technology in three-dimensional cerebral angiography, skull-base clipping techniques, ICG videoangiography, micro-Doppler ultrasound, intraoperative digital subtraction angiography (DSA), neuromonitoring, postoperative diffusion-weighted MRI (DWI), and perioperative care. Era of treatment was not intended to represent a primary bedside predictor variable because significant dependence on the calendar era of treatment would severely limit biological transportability.

PComA-O utilized a unique validation strategy because only 30 patients with preoperative CN III palsy were treated during 2021–2026 and only two patients failed to achieve recovery of function by one year. Therefore, repeated nested resampling, along with corrections for optimism via bootstrapping, comprised our primary internal-validation strategy. The cohort of recently treated patients was utilized solely as a temporal stress test. As such, PComA-O should be viewed as exploratory.

### 2.5. Multilayer Statistical, Machine-Learning, and Artificial-Intelligence Architecture

While the primary goal when evaluating the families of algorithms was to ascertain whether the increased flexibility offered by machine-learning techniques facilitated the detection of reproducible patterns of association beyond those achievable with well-established clinical approaches, we did not seek to emphasize the extent of computational complexity introduced by the novel architectures.

Firstly, the selection of all endpoint models was performed on the basis of structural form. For example, logistic regression was used for binary outcomes; Firth-penalized logistic regression for sparse or separated binary outcomes; proportional-odds logistic regression for ordinal outcomes; and Cox or flexible parametric models for time-to-event data. Furthermore, ridge and elastic-net regularization were utilized during model selection to mitigate multicollinearity in the datasets studied, while simultaneously promoting sparsity in the resultant models’ representation of predictor–outcome relationships. The utilization of restricted cubic spline functions enabled the representation of nonlinear relationships between continuous predictor variables and outcomes without having to discretize the predictor variables.

Bayesian regression was utilized to perform sensitivity analyses and provide estimates of posterior uncertainty for our models.

We investigated a family of nonlinear tabular models that included Random Forest, Extra Trees, XGBoost, LightGBM, and CatBoost. Explainable Boosting Machines (EBMs) permitted interpretable results because they included both nonlinear feature functions and pairwise interactions between features. We evaluated shallow CART models exclusively as examples of compact rule-based comparator models.

Once we had carried out dimensionality reduction through clinician-guided methods, we evaluated TabPFN as a potential modern tabular foundation-model benchmark. Following this, we compared TabPFN with a regularized transformer-based tabular architecture. After verifying that the new models demonstrated better discrimination along with acceptable calibration, resampling stability, temporal behavior, and clinically reasonable interpretative frameworks, we retained only those new models that met these requirements.

Because PComA-O represents a longitudinal dataset of patient information gathered over time, the family of survival-learning models that we developed included elastic-net Cox regression, Random Survival Forests, gradient-boosted survival modeling, and XGBoost accelerated failure-time modeling. Due to limited temporal validation and variability under resampling, we treated neural survival models as experimental and retained them for further evaluation only if they produced consistent predictions under resampling.

When multiple models exhibited comparable performance across endpoints, we employed cross-fitted stacking based only on out-of-sample base-learner predictions. When stacking ensembles displayed better calibration, bootstrap stability, and temporal performance than their respective individual base learners, these stacking ensembles were retained (Table 8).

Global Interpretation of Model Behavior

To facilitate global interpretation of our models’ behavior, we employed several global interpretation tools, including permutation importance, accumulated local effects (ALE), EBM shape functions, partial dependence plots where feasible, and SHAP attribution. Generally, we preferred developing local interpretations from either out-of-sample or temporally withheld predictions. In addition to developing local interpretations of model behavior, we also assessed the ranking stability of features across bootstrap-sampled versions of the model.

Before model development, biologically plausible interactions were predefined. These included PComA–P1 diameter interaction, PComA incorporation–neck width interaction, AChA proximity–neck geometry interaction, retro-dome perforators–aneurysm projection interaction, CN III compression–duration of palsy interaction, CN III grooving–complete versus incomplete palsy interaction, and baseline neurological severity–vascular-risk anatomy interaction. These interactions were regarded as either predictive or hypothesis-generating associations rather than causal associations.

Contributions to Domain Expertise

We measured anatomical contributions through anatomical-domain ablation. Anatomical-domain ablation consisted of measuring how much each previously defined domain contributed individually to the prediction process. Contributions from domains included PComA–P1 dependency, AChA anatomy/geometry/topology, CN III anatomy/morphology, high-resolution morphology, and perforator topology. By examining changes in discrimination, calibration, and decision-curve behavior, we were able to differentiate between PComA-specific information and other general predictors such as age, rupture status, GCS value, aneurysm size, etc.

Predictive Uncertainty

Predictive uncertainty was considered an inherent component of our model outputs. We estimated predictive uncertainty using both bootstrap-sample variability and Bayesian posterior uncertainty. Disagreement among high-performing model families was also utilized as a means of estimating predictive uncertainty. Conformal prediction methodology quantified predictive uncertainty in addition to providing prediction sets or intervals at specified coverage levels rather than providing a single classification by default.

Broad prediction sets that encompassed wide ranges of possible classifications and/or included unusual combinations of predictors were considered indicative of the need for greater caution prior to clinical interpretation and potentially justified model abstention. Model abstention referred to insufficient predictive confidence for generating a reliable model-based prediction and thus necessitated additional expert clinical evaluation.

Converting AI Models to Clinically Applicable Bedside Assessments

All of the complete computational models developed during this study served as discovery platforms from which candidate bedside features were determined according to temporal relevance, bootstrap-sample stability, directionality, rank stability across models, anatomical plausibility, avoidance of redundancy, and practical measurability. Compact point-based scores representing maximal predictive information from each complete AI model were created using RiskSLIM/FasterRisk-type methods incorporating sparse integer optimization. Complete AI models, reduced interpretable models, and sparse bedside scores were evaluated independently. Rather than arbitrarily assigning common-scale representations to score weights, optimal theoretical bounds for score weights were determined through optimization. Post hoc risk assessments were grouped based upon observed calibration behavior and decision-curve behavior; however, these groups were not considered externally validated treatment thresholds.

### 2.6. Model Evaluation and Statistical Analysis

Our models’ overall quality was determined by assessing the models’ discrimination capabilities, calibration, clinical net benefit, interpretability, uncertainty, robustness, and temporal performance. Discrimination was defined as the ability to differentiate between subjects experiencing the endpoint of interest and subjects who did not experience the endpoint. High discrimination rates are necessary but not sufficient to confirm that a model can be utilized in a clinical environment. The models also need to be well calibrated (i.e., not systematically overestimating or underestimating risk), consistent (i.e., demonstrating stable performance across evaluated populations), and capable of accurately predicting outcomes in temporally subsequent cases.

To evaluate how well our models discriminated between individuals with and without the endpoint of interest, we used receiver operating characteristic area under the curve (ROC AUC) and precision–recall area under the curve (PR-AUC). In addition, we utilized other metrics to compare how our models performed under varying classification scenarios: sensitivity (the proportion of individuals with the endpoint of interest who were correctly classified); specificity (the proportion of individuals without the endpoint of interest who were correctly classified); positive predictive value (PPV) (the proportion of individuals classified as having the endpoint of interest who truly had the endpoint of interest); negative predictive value (NPV) (the proportion of individuals classified as not having the endpoint of interest who did not have it); balanced accuracy (a metric that considers both sensitivity and specificity); F1 score (the harmonic mean of precision and recall); and Matthews correlation coefficient (MCC) (a statistic measuring the relationship between predicted and observed binary classifications).

Calibration is defined as how well the predicted probability of an event matches the observed frequency of that event. Calibration was evaluated using Brier score, calibration intercept and slope, integrated calibration error/index, and graphical calibration curves with resampling-based confidence bands. All recalibration parameters were calculated exclusively from the development dataset.

We also evaluated the performance of our models on ordinal outcome scales. For example, for the ordinal PComA-E endpoint, we estimated ordinal concordance, Somers’ Dxy, average absolute ordinal errors, and, as a secondary outcome measure, the proportion of correct mRS classifications.

For models estimating time to recovery for PComA-O, we analyzed their performance using Harrell’s C-index, time-dependent AUC, integrated Brier score, calibration of cumulative recovery probabilities, and agreement between observed and predicted recovery patterns. Additionally, we assessed competing-risk sensitivity using cumulative-incidence methods.

Decision-curve analysis was used to calculate net benefit over clinically relevant ranges of probability. Any threshold values derived from this analysis should never be used as evidence for or against microsurgical intervention or avoidance. Decision-curve thresholds should only be seen as possible prompts for additional anatomical review, vascular imaging, multidisciplinary team discussion, further verification of surgical interventions, or increased postsurgical monitoring. Even if a model demonstrates a positive decision-curve region, a model with modest discrimination or poorly calibrated absolute-risk predictions should not be presumed to be clinically useful solely on that basis.

Each full PComA-CORE model was compared with standard reference models, reduced interpretable models, and sparse scores via paired resampling and bootstrap distributions to analyze performance with respect to discrimination, Brier score, calibration, and clinical net benefit. These comparisons were used to quantify the trade-offs associated with retaining detailed computation versus bedside usability.

Modeling optimism was quantitatively assessed using 2000 bootstrap samples through repeated evaluations of model performance on each bootstrap sample, after which each bootstrap-fitted model was applied to the complete development dataset. The average difference between these two sets of model performance values represented the level of optimism. Additionally, the apparent development performance was adjusted by subtracting the estimated level of optimism from it to determine an optimism-corrected estimate of model performance. We created learning-curve plots at five different percentages of the development dataset (i.e., 20%, 40%, 60%, 80%, and 100%) and graphically illustrated how well our models performed relative to their respective training and validation datasets. We utilized the area under the receiver operating characteristic curve (ROC AUC) to evaluate the PComA-C and PComA-R models. In contrast, we employed concordance indices to assess the performance of the PComA-O survival model and the ordinal PComA-E model.

In addition to analyzing how each model behaved and its clinical utility, we also assessed the transportability of each model across various cohorts. Assessed cohorts included rupture status; adult versus partial or complete fetal posterior circulation; ICA–PComA versus true PComA origin; aneurysm size; age; sex; treatment year; and, within PComA-O, partial versus complete palsy. Special attention was given to assessing performance in the 2021–2026 temporal cohort and performance in anatomically unusual subgroups (Table 9).

Continuous variables were reported as mean ± SD when approximately normally distributed and as median [IQR] when skewed. Categorical variables were reported as counts and percentages. Attention was focused on interpreting both the magnitude of effects and uncertainty/credibility estimates, along with calibration, out-of-sample performance, stability, and clinical relevance, rather than *p*-values alone. Two-sided α levels of 0.05 were conventionally applied for hypothesis testing; exploratory interaction analyses, subgroup analyses, and mechanistic analyses were generally interpreted based on magnitude of effect, uncertainty, consistency, and biological plausibility.

All analyses were performed locally using Python (v3.14.7) and R (v4.6.1). Python packages included NumPy (v2.5.1), pandas (v3.0.5), scikit-learn (v1.9.0), SHAP (v0.52.0), XGBoost (v3.4.0), LightGBM (v4.7.0), CatBoost (v1.2.10), InterpretML (v0.7.8), TabPFN (v8.5.0), PyTorch (v2.13.0), scikit-survival (v0.28.0), MAPIE (v1.5.0), Optuna (v4.9.0), FasterRisk (v0.1.10), and Matplotlib (v3.11.1). R packages included rms (v8.1-1), brglm2 (v1.1.0), logistf (v1.26.1), rstanarm (v2.32.2), brms (v2.23.0), glmnet (v5.0), ordinal (v2026.7-26), pROC (v1.19.0.1), dcurves (v0.5.1), mice (v3.19.0), rpart (v4.1.27), randomForest (v4.7-1.2), qcc (v2.7), and irr (v0.85). These packages supported preprocessing, regression and survival modeling, machine learning, validation, calibration, explainability, uncertainty estimation, score derivation, and statistical inference. Computations were performed on a 64-bit workstation with an Intel Core i7-13620H processor (2.40 GHz; Intel Corporation, Santa Clara, CA, USA) and 16 GB RAM in accordance with institutional data-protection requirements.

## 3. Results

### 3.1. High-Resolution Neurovascular Phenotype and Clinical Outcome Landscape

This study utilized a database of 779 variables that documented extensive variability among 687 microsurgically treated PComA aneurysms. Anatomical relationships (branch incorporation, perforator location, relationships to cranial nerves, etc.), perioperative factors (operative difficulty, tissue-level ischemic injury, etc.), and long-term neurological recovery were all recorded.

Anatomical involvement was frequent. The PComA was directly involved in 143 cases (20.8%) where it was incorporated into the aneurysmal neck, in 51 cases (7.4%) where it was incorporated into the aneurysm sac, and in 118 cases (17.2%) where it was adherent to the aneurysm. The AChA was directly involved in 57 cases (8.3%) where it was incorporated into the neck, in 18 cases (2.6%) where it was incorporated into the sac, and in 62 cases (9.0%) where it was adherent to the aneurysm. Due to their potential impact upon cerebral function when compromised, these types of vessel relationships were evaluated independently of other parameters that typically characterize aneurysm morphology, including aneurysm size and neck width.

Another factor adding to the spatial complexity of the surgical approach to PComA aneurysms was perforator anatomy. In addition to being directly involved in the neck in 105 patients (15.3%), in the sac in 41 (6.0%), or hidden behind the dome of the aneurysm in 127 cases (18.5%), perforators also crossed over the dome in 264 cases (38.4%). A premammillary artery was present in 503 patients (73.2%), with a premammillary origin noted at either the neck or sac level in 49 cases (7.1%) and direct aneurysm adherence in 47 cases (6.8%). Therefore, in many instances, the vascular-preservation challenge inherent to treating a PComA aneurysm extended beyond preserving the parent communicating artery and included a complex network of distributed branch–perforator interactions.

The degree of anatomical interaction between CN III and the aneurysm was equally varied. Anatomical contact between CN III and the aneurysm was documented in 264 patients (38.4%), CN III was grooved in 77 cases (11.2%), and stretched in 62 cases (9.0%); prior to surgery, clinically evident oculomotor palsy was documented in 175 patients (25.5%). Because anatomical contact with CN III exceeded overt clinical dysfunction, anatomical nerve interaction and clinical palsy were maintained as separate measures for use in modeling subsequent recovery.

Operative behavior varied across the entire range of anatomical variation described above. A high-complexity operative phenotype was identified in 122 patients (17.8%). Temporary clipping was performed in 384 patients (55.9%), intraoperative rupture of the aneurysm occurred in 85 patients (12.4%), and composite technical success was achieved in 534 patients (77.7%), while composite technical-safety failure occurred in 112 patients (16.3%). Since temporary clipping, intraoperative rupture, and composite technical success/safety failure represent related but distinct dimensions of operative performance, they were each analyzed separately.

Complete obliteration of the aneurysm after microsurgical clipping was documented in 551 of 589 patients with assessable early obliteration status (93.5%), with a residual neck documented in 30 cases (5.1%) and a residual sac documented in 8 cases (1.4%). Preserved flow through the PComA/PCA was documented in 667 of 687 patients (97.1%), preserved AChA flow in 676 of 687 patients (98.4%), and preserved perforator flow in 663 of 687 patients (96.5%).

While >95% of patients demonstrated preserved macroscopic arterial flow postoperatively, procedure-related ischemic injuries occurred in 87 patients (12.7%), while overt branch-preservation failures occurred in 28 patients (4.1%). Fifteen patients experienced both procedure-related ischemic injury and overt branch-preservation failure.

Procedure-related ischemic injuries often affected multiple territories. Lesions affecting PCA territories occurred in 28 patients (32.2% of procedure-related ischemic injuries), lesions affecting perforator territories occurred in 22 patients (25.3%), lesions affecting AChA territories occurred in 18 patients (20.7%), lesions affecting internal-capsular territories occurred in 14 patients (16.1%), lesions affecting thalamic territories occurred in 11 patients (12.6%), lesions affecting hypothalamic territories occurred in six patients (6.9%), and lesions affecting premammillary territories occurred in four patients (4.6%). Multifocal patterns of injury were observed in 16 patients (18.4%).

Most postoperative tissue-level ischemic injuries occurred even though major-vessel flow had been preserved postoperatively. More specifically, many procedure-related ischemic injuries occurred despite preserved PComA flow, preserved AChA flow, and preserved perfusion through visualized perforators. These findings supported our decision to treat tissue-level procedure-related injury and overt branch-preservation failure as two separate but partially overlapping concepts.

Modified Rankin Scale evaluations at 90 days were available for 635 patients. Favorable outcomes, defined as mRS ≤ 2, were observed in 468 patients (73.7%), while unfavorable outcomes, defined as mRS > 2, were observed in 167 patients (26.3%). The ordinal distribution of Modified Rankin Scale scores was as follows: mRS = 0 in 148 patients; mRS = 1 in 165 patients; mRS = 2 in 155 patients; mRS = 3 in 89 patients; mRS = 4 in 26 patients; mRS = 5 in five patients; and mRS = 6 in 47 patients. By 12 months, Modified Rankin Scale scores were available for 531 patients, with mRS ≤ 2 observed in 427 patients and mRS > 2 observed in 104 patients.

Patients who entered surgery with preoperative oculomotor palsy exhibited a distinct recovery pattern. Recovery progressed rapidly early after surgery, followed by gradual resolution of remaining deficits. Complete recovery from oculomotor palsy was documented in 112 of 175 patients by 90 days, in 142 patients by six months, in 155 patients by 12 months, and in 156 patients by 24 months postoperatively. The median time to first documented improvement in oculomotor function was approximately 28 days, while the median time to complete recovery was approximately 61 days.

The median time to recovery of specific aspects of oculomotor function also varied: median time to recovery for ptosis was approximately 43 days; median time to recovery for pupillary dysfunction was approximately 48 days; and median time to recovery for diplopia was approximately 57 days.

In contrast to those with complete recovery of oculomotor function by 12 months postoperatively, those without complete recovery had persistent ptosis, diplopia, pupillary dysfunction, and/or ophthalmoplegia.

Recurrence or regrowth was documented during available follow-up in 13 patients (1.9%), while 14 patients (2.0%) underwent re-intervention during available follow-up.

Primary neurovascular, technical, ischemic, functional, and CN III outcomes are provided below in Table 10.

### 3.2. PComA-C: Preoperative Prediction of Microsurgical Complexity

The primary purpose of developing PComA-C was to determine microsurgical difficulty prior to making an incision. This included six elements of technical difficulty as follows: skull-base exposure; clip reconstruction; cumulative temporary occlusion time > 10 min; ≥3 clip repositionings; flow rescue; and intraoperative rupture necessitating escalation of vascular control. At least two complexity factors were required, while any flow-rescue maneuver or ≥3 clip repositionings alone fulfilled the criteria. Postoperative complication rates and postoperative functional outcomes were not included in defining the phenotype.

In total, among the 564 patients in the development cohort, 100 (17.7%) met the PComA-C phenotype. There were 22 (17.9%) of the 123 patients in the same-center temporal cohort from 2021–2026 who met the phenotype. Although there have been significant advances in both microsurgical techniques and methods of intraoperative verification over the study period, the percentage of high-complexity cases remained relatively stable.

Statistical models using traditional methods identified a considerable amount of predictive signal. The internal area under the receiver operating characteristic curve (AUC) for logistic regression was 0.842, and the temporal AUC was 0.851. Firth logistic regression also provided AUCs of 0.849 and 0.858 for the internal and temporal analyses, respectively. Discrimination was improved further by elastic-net regularization, which resulted in an internal AUC of 0.873 and temporal AUC of 0.886.

Higher discrimination was obtained through tree-based models. The Random Forest model resulted in a temporal AUC of 0.907, Extra Trees in an AUC of 0.918, XGBoost in an AUC of 0.931, LightGBM in an AUC of 0.922, CatBoost in an AUC of 0.943, EBM in an AUC of 0.895, TabPFN in an AUC of 0.884, and the regularized tabular transformer model in an AUC of 0.872. The cross-fitted stacking method produced an internal out-of-fold ROC AUC of 0.918 and a same-center temporal AUC of 0.932. Since the stacked ensemble did not result in a better temporal AUC than the anatomy-informed CatBoost model, it was not retained as the final model.

The final anatomy-informed CatBoost model produced a five-fold out-of-fold ROC AUC of 0.911 (95% CI, 0.880–0.939), PR-AUC of 0.736, and Brier score of 0.0808. In the chronologically separated 2021–2026 same-center cohort, ROC AUC was 0.943 (95% CI, 0.894–0.978), PR-AUC was 0.800, and Brier score was 0.078. Temporal calibration resulted in a slope of 1.02 and intercept of −0.43, indicating modest overestimation of absolute risk. Development-derived recalibration parameters slightly reduced the temporal Brier score to 0.077.

The temporal AUC of 0.943 represents a very high level of discrimination for operative complexity and therefore requires cautious interpretation. All predictors for the model were restricted to information available before incision, and the 2021–2026 cohort was isolated from feature selection, data preprocessing, hyperparameter tuning, threshold determination, and calibration. However, PComA-C predicts a registry-defined high-complexity operative phenotype within the same center, and its performance may partially reflect center-specific definitions of operative complexity, surgical strategy, measurement processes, and expertise. Therefore, the observed temporal performance should not be considered proof of portability outside the originating center. Residual information leakage or center-specific coupling between anatomical evaluation and operative behavior also cannot be completely excluded.

At the threshold determined solely from development-cohort data, sensitivity was 0.842, specificity was 0.886, PPV was 0.631, NPV was 0.967, balanced accuracy was 0.864, F1 score was 0.721, and MCC was 0.708. The high observed NPV indicated good differentiation of lower-complexity operations within this dataset; however, predictive values are dependent upon event prevalence and setting and require independent external evaluation.

Decision-curve analysis indicated positive temporal net benefit across probability thresholds of 10–30%, resulting in net-benefit values of 0.150, 0.135, 0.122, 0.100, and 0.091 at threshold values of 10%, 15%, 20%, 25%, and 30%, respectively. These data represent same-center decision-analytic behavior and should not be interpreted as clinically validated treatment thresholds.

Value of High-Resolution Anatomical Detail

**Figure 1:**
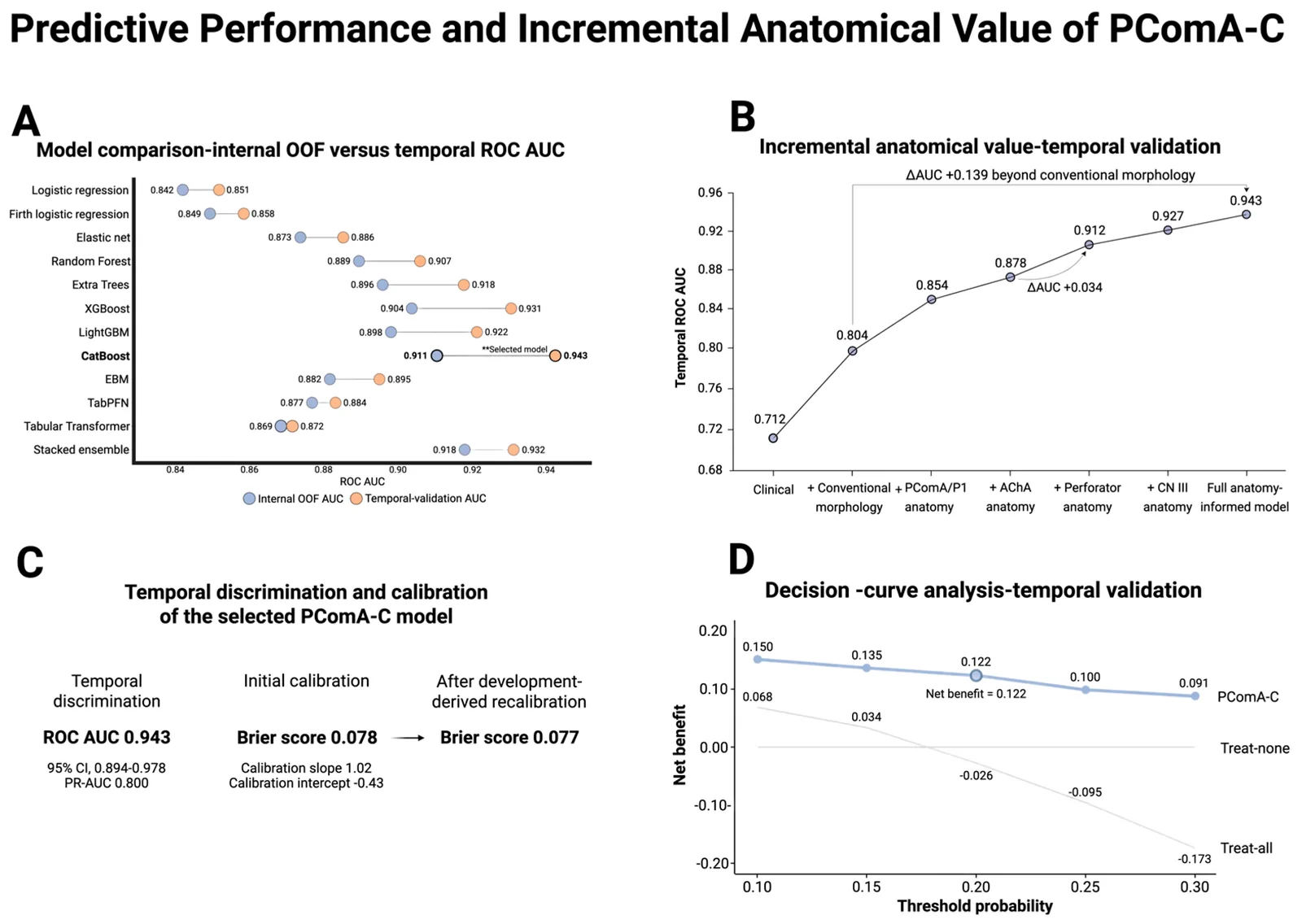
Predictive performance and incremental anatomical value of PComA-C. (**A**) Internal out-of-fold and temporal-validation ROC AUC across candidate models, with CatBoost retained as the final architecture. (**B**) Domain-ablation analysis showing improvement in temporal AUC from 0.712 with clinical variables alone to 0.943 with the full anatomy-informed model (ΔAUC +0.139 beyond conventional morphology). (**C**) Temporal discrimination and calibration of the selected CatBoost model. (**D**) Decision-curve analysis demonstrating positive net benefit across threshold probabilities of 0.10–0.30. AChA, anterior choroidal artery; CN III, oculomotor nerve; OOF, out-of-fold; PComA, posterior communicating artery; PR-AUC, area under the precision–recall curve; ROC AUC, area under the receiver operating characteristic curve. ** Indicates the model retained as the final PComA-C architecture.

A prespecified domain–ablation analysis quantified how much each successive layer of anatomical detail contributed beyond what could be gained from the preceding layers. A clinical-only model based on age, sex, rupture status, Hunt–Hess grade, and WFNS grade achieved a temporal AUC of 0.712. Adding conventional aneurysm morphology increased the AUC to 0.804; adding PComA/P1 anatomy increased the AUC to 0.854; adding AChA anatomy increased the AUC to 0.878; adding perforator anatomy increased the AUC to 0.912; adding CN III anatomy increased the AUC to 0.927; and adding all anatomical layers increased the AUC to 0.943. The contributions to discrimination made by each layer are summarized graphically in Figure 1.

After accounting for conventional aneurysm morphology, addition of PComA-specific neurovascular anatomy increased temporal AUC from 0.804 to 0.943. Perforator anatomy alone increased temporal AUC from 0.878 to 0.912. Together, these results indicate that improvements in discrimination within this dataset were associated with the additional anatomical detail represented by the PComA/P1, AChA, perforator, and CN III domains.

Important features included neck width, dome-to-neck ratio, parent ICA curvature, PComA sac adherence/neck incorporation, P1-origin perforators, aspect ratio, aneurysm dimensions, AChA proximity, hypothalamic/perforator-risk anatomy, and CN III relationships. Overall, these results indicate that the PComA-C predictive signal was distributed across reconstructive geometry, parent-vessel configuration, spatial constraints of the operative corridor, branch incorporation, and perforator topology rather than aneurysm size alone.

### 3.3. PComA-R: Neurovascular Preservation and Procedure-Related Ischemic Risk

The PComA-R study investigated the likelihood of experiencing ischemic injury due to surgical interventions. It defined the outcome studied according to one of two criteria: (a) new DWI lesions detected in the territories of the posterior communicating artery (PComA)/P1/posterior cerebral artery (PCA)/anterior choroidal artery (AChA)/perforators, or (b) postoperative neurological deficits attributable to ischemic injury caused by the surgical procedure. Preoperative lesions and delayed cerebral ischemia or vasospasm that could not be directly associated with the surgical procedure were excluded. When appropriate, MRI-DWI was acquired within 48–72 h after the operation. CT scans were obtained when MRI was contraindicated or when additional assessment of symptoms was required.

Of the 564 patients included in the development group, 69 (12.2%) met the outcome criteria. Of the 123 patients in the same-center temporally separated cohort from 2021–2026, 18 (14.6%) met the outcome criteria. Predictive modeling of ischemic injury related to surgical interventions was substantially more difficult than predictive modeling of surgical procedure complexity.

Using temporal area under the receiver operating characteristic curve (AUROC), the predictive models tested in the study ranged from 0.648 for logistic/Firth regression to 0.703 for CatBoost, while stacked predictions achieved an AUROC of 0.698. For the most part, differences between models were modest.

The CatBoost model used to evaluate PComA-R exhibited an internal out-of-fold AUROC of 0.681 (95% CI, 0.615–0.744), precision-recall area under the curve (PR-AUC) of 0.212, and Brier score of 0.112. In the same-center temporal evaluation cohort, AUROC was 0.703 (95% CI, 0.565–0.832), PR-AUC was 0.314, and Brier score was 0.118. Raw temporal calibration parameters exhibited an intercept of −0.53 and a slope of 0.54, demonstrating imperfect portability of absolute-risk estimates. Temporal recalibration based on development-derived parameters improved probability estimates only slightly, as the temporal Brier score remained close to 0.118.

Therefore, PComA-R showed modest discriminative capacity and imperfect absolute-risk calibration overall. Given that there were only 87 ischemic events throughout the entire dataset, together with a potentially large number of candidate anatomical predictors and wide temporal confidence intervals, residual overfitting remains possible despite regularization, nested cross-validation, and temporal separation.

Accordingly, PComA-R should be regarded primarily as an exploratory representation of relative anatomical susceptibility to procedure-related ischemic injury rather than as a stand-alone clinical prediction instrument.

At the predetermined cut-point, sensitivity was approximately 66.8%, while specificity was approximately 61.4%. Positive predictive value (PPV) was 19.5%, while negative predictive value (NPV) was 93.5%. Balanced accuracy was 64.1%, F1 score was 0.302, and Matthews correlation coefficient (MCC) was 0.238. Importantly, the low PPV indicates that a positive PComA-R classification does not reliably predict that a patient will experience procedure-related ischemia. Likewise, although the NPV was relatively high, this finding should be interpreted in the context of the low event prevalence and requires independent external validation.

Decision-curve analysis also indicated that PComA-R has limited clinical utility in its present form. Net benefit at a probability threshold of 10% was approximately 0.058; at 15%, approximately 0.052; at 20%, approximately 0.039; at 25%, approximately 0.016; and at 30%, approximately −0.004. While some degree of net benefit was observed at lower probability thresholds, these findings do not support the use of PComA-R as a stand-alone clinical decision aid.

**Figure 2:**
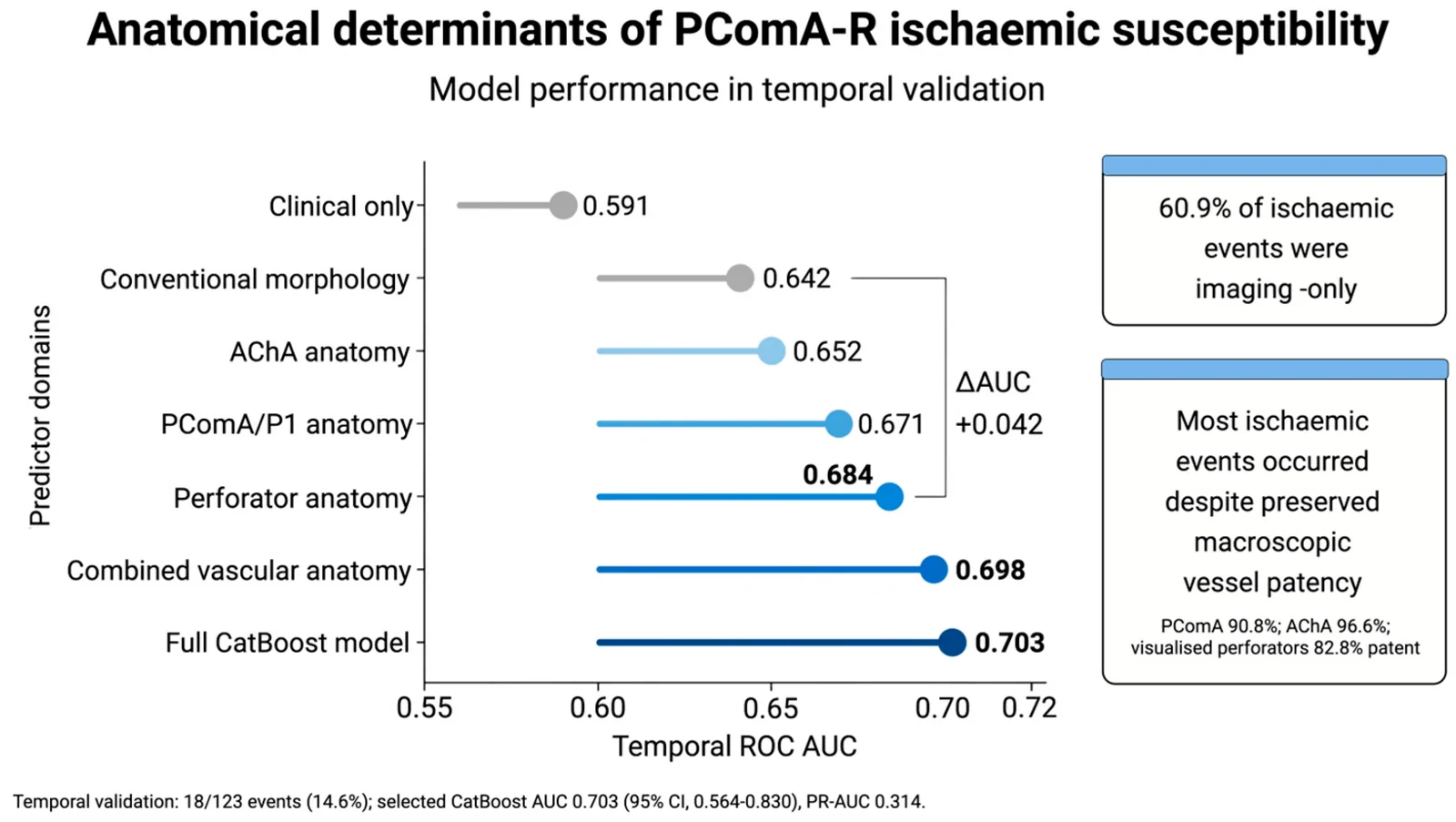
Domain-specific prediction of PComA-R ischemic susceptibility. Temporal discrimination across clinical, morphological, and vascular-anatomical predictor domains is shown, with perforator anatomy outperforming conventional morphology (AUC 0.684 vs. 0.642) and the full CatBoost model reaching an AUC of 0.703. Most procedure-related ischemic events were imaging-only and occurred despite preserved macroscopic vessel patency. AChA, anterior choroidal artery; AUC, area under the receiver operating characteristic curve; PComA, posterior communicating artery; PR-AUC, area under the precision–recall curve.

Examining each domain within PComA-R individually revealed an AUROC of 0.591 for clinical characteristics alone and an AUROC of 0.642 for conventional morphology alone. Examination of PComA/P1 anatomy yielded an AUROC of 0.671; examination of AChA anatomy yielded an AUROC of 0.652; and examination of perforator anatomy yielded an AUROC of 0.684. Combining all relevant vascular anatomy increased discrimination to an AUROC of 0.698; however, combining all relevant vascular anatomy with the complete CatBoost architecture produced only a slight additional increase in discrimination to an AUROC of 0.703. The contributions of vascular anatomy to PComA-R prediction of procedure-related ischemia are summarized in Figure 2, together with their relationship to the imaging phenotype associated with procedure-related ischemia.

These findings indicate that perforator and small-vessel topology contributed additional predictive information beyond that identified through conventional aneurysm morphology; however, they do not demonstrate that perforator anatomy accounts for all pathophysiological mechanisms underlying procedure-related ischemia. Rather, PComA-R appears to represent a multifactorial susceptibility phenotype incorporating elements of small-vessel anatomy, procedural injury, hemodynamics, and other perioperative influences.

Supporting evidence for this interpretation was present in the postoperative phenotype: many imaging-defined ischemic lesions were clinically silent, and many occurred despite apparent preservation of major-vessel patency and/or perfusion through visualized perforators. Thus, these findings indicate that PComA-R provides a framework for investigating mechanisms of procedure-related ischemic injury; however, its current performance is insufficient to support its use as an independent clinical decision tool.

### 3.4. PComA-O: Longitudinal Prediction of Oculomotor Recovery

Patients whose CN III palsies predated surgery were defined as having achieved complete oculomotor function recovery when each of the three components of their palsy—ptosis, extraocular movement deficits, and pupillary dysfunction—was restored to either the pre-palsy state or a normal state. Of the 175 patients in this study, 155 (88.6%) returned to a fully functional state by 12 months after surgery. Therefore, 20 patients (11.4%) continued to have some degree of oculomotor dysfunction.

Oculomotor function recovery was associated with numerous clinical and anatomical factors. Recovery was observed in 94 (95.0%) of 99 patients with partial CN III palsy compared with 61 (80.3%) of 76 patients with complete palsy. Likewise, recovery was observed in 98 (96.1%) of 102 patients without pupillary dysfunction compared with 57 (78.1%) of 73 patients with pupillary dysfunction.

Similarly, structural deformation of CN III was associated with the likelihood of recovery. In patients in whom CN III exhibited grooving, complete recovery was observed in 34 (65.4%) of 52 patients. Conversely, in patients with no CN III grooving, complete recovery was observed in 121 (98.4%) of 123 patients. While the separation between groups in recovery rate according to simple aneurysm–nerve contact was much smaller, complete recovery was observed in 98 (90.7%) of 108 patients with documented contact and 57 (85.1%) of 67 patients without documented contact.

A major difference existed in the median duration of preoperative CN III palsy between patients who experienced complete recovery and those who did not experience complete recovery. For patients experiencing complete recovery, the median preoperative palsy duration was 46.2 days (IQR, 21.5–82.4). Conversely, for patients who failed to experience complete recovery, the median duration was 189.4 days (IQR, 112.8–356.2; *p* < 0.001).

Survival Models and Fixed-Horizon Estimates

Within the 145-patient development-stage PComA-O cohort, evaluation of the time-to-event models showed that the C-index for Cox regression was 0.812, while the C-index for elastic-net Cox regression was 0.824. Additionally, Random Survival Forest had a C-index of 0.841, gradient-boosted survival modeling had a C-index of 0.846, and XGBoost accelerated failure-time modeling had a C-index of 0.858. The integrated Brier score was 0.118.

For time-dependent AUC values, the AUC at 90 days postoperatively was 0.874, the AUC at six months postoperatively was 0.889, and the AUC at one year postoperatively was 0.902. These AUC values indicate a high degree of discrimination in the derivation-stage analyses. However, these results cannot be considered evidence of temporal or external validation.

Evaluating eight machine-learning models using a one-year fixed horizon, penalized logistic regression yielded an AUROC of 0.861, Random Forest yielded an AUROC of 0.858, Extra Trees yielded an AUROC of 0.885, Explainable Boosting Machine (EBM) yielded an AUROC of 0.871, TabPFN yielded an AUROC of 0.848, LightGBM yielded an AUROC of 0.892, CatBoost yielded an AUROC of 0.888, and XGBoost yielded an AUROC of 0.902. Cross-validation estimates of out-of-fold discrimination within the development dataset indicated that discrimination appeared somewhat lower for each model, with an overall average discrimination estimate across models of approximately 0.83.

Utilizing landmark analysis with XGBoost to derive a one-year AUROC estimate resulted in an estimated AUROC of 0.902 (95% CI, 0.828–0.960), PR-AUC of 0.666, Brier score of 0.075, and calibration slope of 0.96. Similar to the previous estimates, these represent internal evaluation and derivation-stage performance for estimating outcomes related to PComA-O. Therefore, independent temporal and external validation is required before clinically meaningful thresholds can be established for use in clinical decision-making.

Sequential Domain Analysis

Sequential domain analysis demonstrated improvements in discrimination as additional predictor domains were included in the analyses. When comparing patients with partial versus complete palsy together with pupil involvement, AUROC was 0.754. Adding palsy duration to the analysis increased AUROC to 0.831. Further adding direct CN III anatomy increased AUROC to 0.874. Incorporating aneurysm morphology increased AUROC to 0.891. Incorporating the entire XGBoost model increased AUROC to 0.902.

**Figure 3:**
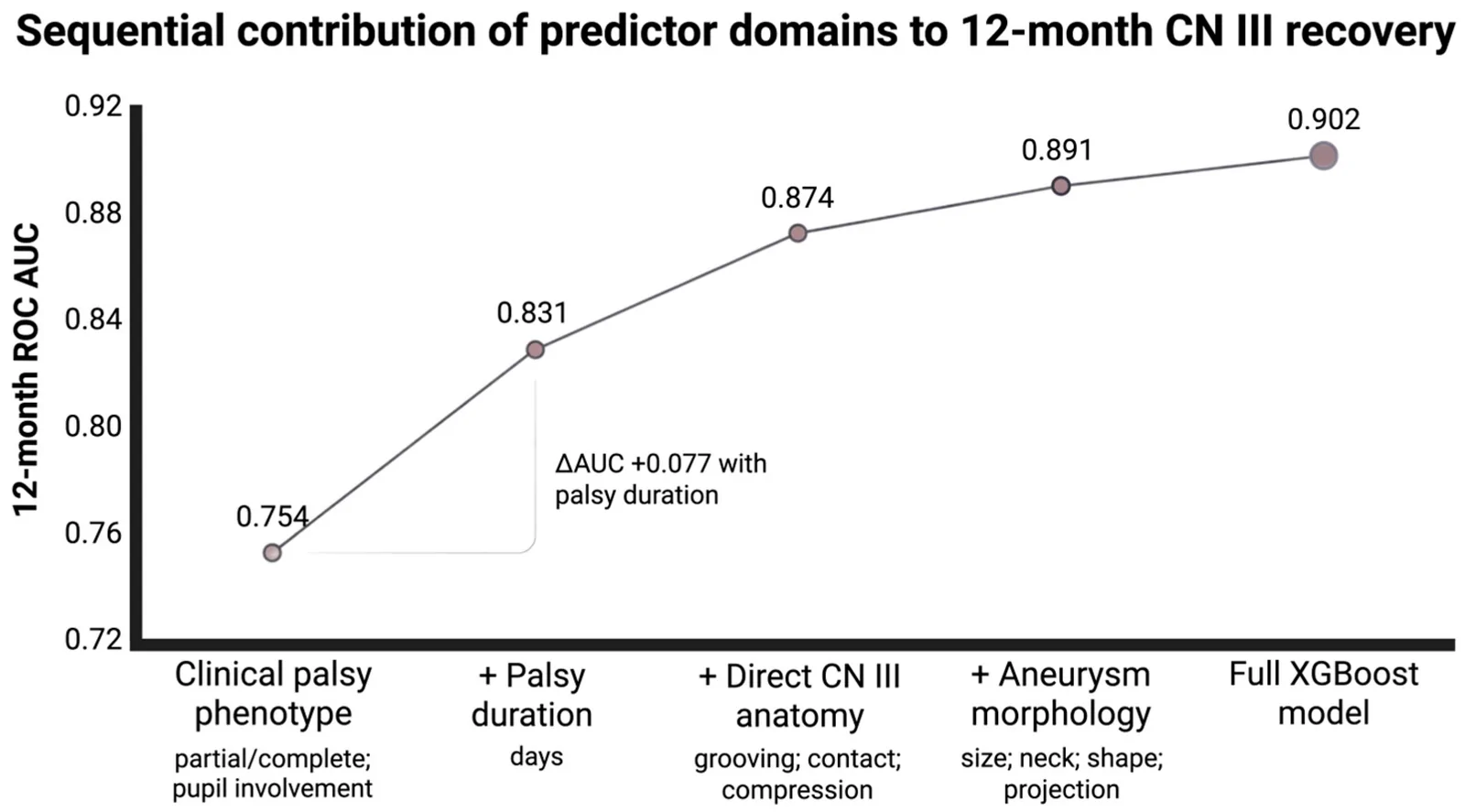
Sequential contribution of predictor domains to 12-month PComA-O oculomotor recovery. The figure demonstrates the progressive increase in discrimination obtained by sequentially incorporating clinically and anatomically distinct predictor domains. A model based on clinical palsy phenotype alone achieved an AUC of 0.754. Addition of preoperative palsy duration increased the AUC to 0.831 (ΔAUC +0.077), followed by direct CN III anatomy to 0.874, aneurysm morphology to 0.891, and the full XGBoost model to 0.902. The pattern illustrates the incremental predictive value of temporal and anatomical information beyond conventional clinical characterization of CN III palsy.

Each increase represents the sequential contribution to predictive discrimination made by progressively larger predictor domains. The incremental contribution of each predictor domain is illustrated in Figure 3.

These estimates represent internally derived, development-stage performance rather than measures of temporal or external validity.

Interaction Analysis

Interaction analysis investigated whether specific combinations of predictors were associated with persistent oculomotor dysfunction. In examining whether patients with both complete palsy and CN III grooving were less likely to experience complete recovery by 12 months after surgery, we found that among the 28 patients exhibiting both characteristics, 14 (50.0%) failed to recover completely by 12 months postoperatively (OR = 8.34; 95% CI, 3.12–22.28; *p* < 0.01). Similarly, among the 18 patients exhibiting grade 3–4 compression and palsy duration > 90 days, 12 (66.7%) failed to recover completely by 12 months after surgery (OR = 12.64; 95% CI, 4.42–36.18; *p* < 0.01).

These findings provide evidence suggesting possible interaction effects between clinical duration and structural severity contributing to persistent dysfunction. These findings are regarded as hypothesis-generating and do not support causal inference.

Temporal Evaluation

Due to the limited number of patients in the same-center temporal cohort with preoperative CN III palsy (*n* = 30), and because only two patients failed to experience complete recovery by one year postoperatively, we were unable to perform reliable standalone temporal validation for PComA-O. Temporal AUC values derived from only two non-recovery events would be statistically unstable and potentially misleading.

As such, PComA-O should be viewed as an internally derived, derivation-stage model and therefore requires independent temporal and external validation to determine the clinical applicability of risk thresholds or clinical utility prior to adoption in clinical practice.

### 3.5. PComA-E: Prediction of the Complete 90-Day Functional Outcome Spectrum

In addition to the PComA-C evaluation, PComA-E was also analyzed using a separate dataset of 635 patient records with complete 90-day modified Rankin Scale (mRS) data. In order to retain all seven ordinal mRS categories and not reduce functional outcome to a single dichotomous variable, an ordinal modeling approach was utilized. The ordinal model yielded a concordance index of 0.866 and Somers’ Dxy of 0.732. Additionally, other metrics used to evaluate the ordinal model included a quadratic-weighted kappa statistic of 0.634, ranked probability score (RPS) of 0.184, and average absolute ordinal error of 0.87 mRS points.

Using the full machine-learning framework to analyze the secondary outcome of mRS > 2, elastic-net regression generated internal and same-center temporal receiver operating characteristic area under the curve (ROC AUC) values of 0.883 and 0.878, respectively. Temporal AUC values obtained with Random Forest were 0.861; Extra Trees, 0.864; XGBoost, 0.869; LightGBM, 0.866; CatBoost, 0.871; EBM, 0.854; and TabPFN, 0.841.

Due to its superior performance and relative simplicity, the elastic-net regression model was selected as the final model. Using internal cross-validation, the AUC obtained was 0.883 (95% CI, 0.851–0.912). The precision–recall area under the curve (PR-AUC) was 0.758. When the chronologically separated 2021–2026 same-center cohort was evaluated, the AUC was 0.878 (95% CI, 0.771–0.959). The PR-AUC was 0.759. The uncalibrated Brier score for this cohort was 0.112.

Although the model maintained similar discriminatory performance across the two cohorts, as reflected by the similar AUC values, its absolute-risk estimation differed. Specifically, the calibration intercept and slope for the 2021–2026 cohort were approximately −1.09 and 1.26, respectively, indicating imperfect calibration of predicted risks relative to observed outcomes in this latter group. However, application of the development-derived recalibration parameters resulted in a substantial reduction in the temporal Brier score to 0.087 and improved calibration, with the calibration intercept and slope becoming approximately −0.21 and 1.03, respectively.

For the operational threshold established during the development phase of PComA-E, temporal sensitivity was 82.8%; specificity was 78.4%; positive predictive value (PPV) was 59.6%; negative predictive value (NPV) was 92.4%; balanced accuracy was 80.6%; F1 score was 0.693; and Matthews correlation coefficient (MCC) was 0.609.

Decision-curve analysis indicated positive temporal net benefit for predicted-risk thresholds ranging between 10% and 30%. More specifically, at thresholds of 10%, 15%, 20%, 25%, and 30%, the corresponding net-benefit values were 0.137, 0.130, 0.103, 0.108, and 0.104, respectively. It is important to note that these decision-curve results reflect same-center temporal behavior and are not intended to be interpreted as externally validated treatment thresholds.

Incremental Value of High-Resolution Anatomical Detail for Global Functional Outcome

Using age, NIHSS, GCS, Hunt–Hess grade, and baseline mRS as input variables in the prediction model resulted in a temporal AUC of 0.856. Adding conventional aneurysm morphology to the input variables increased the AUC to 0.866. The addition of PComA-specific anatomy increased the AUC to 0.872. The addition of the remaining model variables increased the AUC to 0.878.

Unlike PComA-C, where detailed local anatomy contributed substantially to incremental discrimination in predicting microsurgical complexity, high-resolution anatomical information added relatively little incremental discrimination beyond that captured by baseline neurological severity in predicting global functional outcome. To compare behavior across all four PComA-CORE domains, Table 11 provides a summary of performance metrics across several widely used statistical, machine-learning, and AI approaches and their corresponding validation performance metrics.

**Figure 4:**
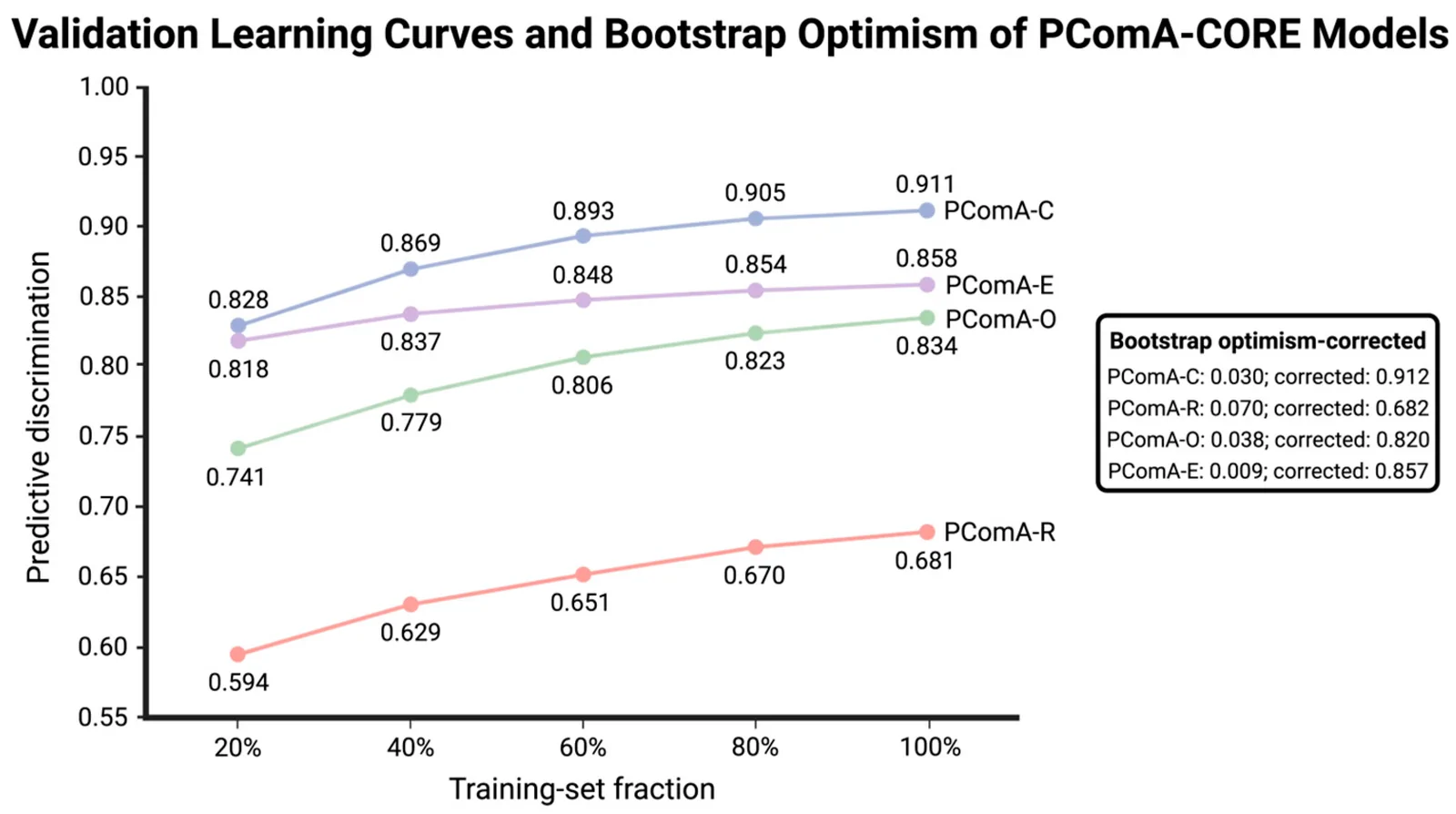
Validation learning curves and bootstrap optimism across the four PComA-CORE components. Validation discrimination is shown across progressively increasing proportions of the corresponding development datasets (20%, 40%, 60%, 80%, and 100%). AUROC was used for PComA-C and PComA-R, whereas concordance indices were used for PComA-O and the primary ordinal PComA-E model. Bootstrap optimism and the corresponding optimism-corrected performance estimates are displayed alongside the learning curves. PComA, posterior communicating artery; AUROC, area under the receiver operating characteristic curve.

Bootstrap analysis evaluated potential optimism within each model. Estimated optimism values were 0.030 for PComA-C, 0.070 for PComA-R, 0.038 for PComA-O, and 0.009 for PComA-E; the estimated optimism-corrected discrimination values were 0.912 for PComA-C, 0.682 for PComA-R, 0.820 for PComA-O, and 0.857 for PComA-E. With regard to the PComA-C and PComA-R models, the estimated optimism-corrected discrimination values were very close to their respective internal out-of-fold AUROCs (0.911 for PComA-C and 0.681 for PComA-R). As would be expected given the reduced number of events supporting the outcome for this model, the higher estimated optimism for PComA-R is reflective of its lower discrimination value compared with the other three models. The learning-curve evaluation also showed increasing validation performance as the amount of data used for training grew (Figure 4), or as the “effective” development sample size increased. Specifically, the PComA-C validation AUROC increased from 0.828 when using 20% of the development sample size to 0.911 when using all 100% of the development sample size. Similarly, the PComA-R validation AUROC increased from 0.594 to 0.681. In addition, the validation C-indices for both PComA-O and PComA-E improved from 0.741 to 0.834 and from 0.818 to 0.858, respectively. Overall, the learning-curve evaluations indicate that there was more stability in both the PComA-C and PComA-E models than in either the PComA-R or PComA-O models.

### 3.6. Uncertainty-Aware Prediction and Anatomical Out-of-Distribution Behavior

The nonconformity score threshold determined by the training dataset was used without modification in the temporal cohort. Thus, conformal prediction maintained explicit ambiguity in the classifications instead of always providing one prediction per patient.

Empirical temporal coverage for PComA-C was 92.7% (114/123), with an average predicted set size of 1.06. The predicted set contained only one class for 116 of the 123 patients (94.3%), while two classes were provided for 7 of the 123 patients (5.7%). No empty prediction sets existed.

Empirical temporal coverage for PComA-R was 91.1% (112/123), with an average predicted set size of 1.13. The predicted set contained only one class for 107 of the 123 patients (87.0%), while two classes were provided for 16 of the 123 patients (13.0%). No empty prediction sets existed.

Empirical temporal coverage for PComA-E was 92.3% (108/117), with an average predicted set size of 1.20. The predicted set contained only one class for 94 of the 117 patients (80.3%), while two classes were provided for 23 of the 117 patients (19.7%). No empty prediction sets existed.

Ambiguity in predictions was significantly higher when the anatomical configurations were less common, such as true PComA origin, complete fetal PCA anatomy, absence or hypoplasia of P1, very large aneurysms, projections that did not fit typical models, and complex retro-dome perforator anatomy. Since this analysis was exploratory and not a formal assessment of anatomical out-of-distribution behavior, uncertainty-aware predictions can be considered an additional method for identifying those cases in which the model’s estimate is less specific and therefore associated with greater uncertainty in anatomically atypical patients.

### 3.7. Intraoperative Verification and the Transition from Anatomical Risk to Tissue Injury

The registry allowed intraoperative verification and rescue to be examined as an intermediary phase between preoperative anatomical risk factors and postoperative tissue-level injury.

Of the 41 ICG examinations that revealed abnormalities, corrective intervention was undertaken in each case, with restoration of flow confirmed and no evidence of postoperative infarction. Conversely, among the 18 abnormal ICG studies in which corrective action failed to restore flow completely or was deemed ineffective, postoperative infarction was observed. Neither of the above pathways accounted for all 86 abnormal ICG studies; some combination of revision, determination of flow restoration, and postoperative imaging outcome occurred in the remaining 27 abnormal ICG studies. Similarly, 19 patients experienced procedure-related ischemia despite having a previously unremarkable ICG study.

Micro-Doppler ultrasonography was employed in 348 patients (50.7%). Of the studies conducted using micro-Doppler ultrasonography, 58 (16.7%) were found to have abnormalities. Revision was conducted in 47 of those 58 abnormal studies (81.0%); documentation of restored flow occurred in 42 of those 47 revised cases (89.4%).

DSA was utilized during surgery in 127 patients (18.5%). The results of intraoperative DSA in this group indicated that there were 18 abnormal examinations (14.2%); these included eight with a small residual aneurysm and ten with compromised branches. A total of 16 (88.9%) of those 18 abnormal examinations underwent intraoperative revision.

Visualization through endoscopy was used in 94 patients (13.7%) and identified previously unrecognized issues associated with either perforators or branches in 11 patients (11.7%). Modification of the clip construct occurred based upon identification of issues through endoscopic visualization in nine (81.8%) of those 11 instances.

Neuromonitoring was used in 263 patients (38.3%). Significant changes were observed in neuromonitoring recordings in 42 patients (16.0%) among those undergoing neuromonitoring. Of those significant changes noted in neuromonitoring recordings, 31 (73.8%) returned to baseline after intervention.

These observational relationships do not indicate that intraoperative verification or rescue was responsible for producing the subsequent clinical outcomes. Rather, they demonstrate an intermediary phenotypic expression representing the transition from preoperative anatomical risk factors through intraoperative detection and corrective actions taken during surgery to the eventual development of tissue-level injury. Additionally, these findings demonstrate that macroscopic assessment of flow may not necessarily exclude procedure-related ischemia.

### 3.8. Reproducibility of High-Resolution Anatomical Phenotyping

High-resolution anatomical phenotyping is crucial for interpreting some of the PComA-CORE predictor features. Therefore, we evaluated inter-observer agreement in a random sample of 142 patients (20.7% of the cohort) whose images were reviewed by two independent observers using a blinded approach.

The results from Cohen’s kappa test indicated strong to near-perfect inter-rater agreement for fetal PCA configuration (κ = 0.87; 95% CI, 0.81–0.92), PComA incorporation (κ = 0.84; 95% CI, 0.78–0.90), AChA incorporation (κ = 0.79; 95% CI, 0.72–0.86), perforator incorporation (κ = 0.77; 95% CI, 0.68–0.85), and aneurysm projection (κ = 0.77; 95% CI, 0.69–0.84). The weighted kappa test showed high inter-rater agreement for CN III compression grade (weighted κ = 0.82; 95% CI, 0.74–0.89) and CN III grooving (weighted κ = 0.83; 95% CI, 0.75–0.91).

For continuous anatomical measurements, the intraclass correlation coefficient for absolute agreement (ICC) ranged from good to excellent as follows: PComA diameter (ICC = 0.89; 95% CI, 0.84–0.94), P1 diameter (ICC = 0.88; 95% CI, 0.82–0.92), AChA distance (ICC = 0.81; 95% CI, 0.74–0.88), aneurysm–CN III distance (ICC = 0.85; 95% CI, 0.78–0.91), neck width (ICC = 0.91; 95% CI, 0.87–0.95), and carotid–oculomotor window (ICC = 0.78; 95% CI, 0.69–0.86).

Finally, when examining derived composite scores from the previous variables, the ICC values ranged from good to excellent as follows: PComA-C (ICC = 0.89; 95% CI, 0.82–0.94), PComA-R (ICC = 0.79; 95% CI, 0.71–0.87), PComA-O (ICC = 0.86; 95% CI, 0.79–0.93), and PComA-E (ICC = 0.92; 95% CI, 0.87–0.96).

Overall, these findings show strong to excellent levels of reproducibility for the major anatomical constructs used in the current research setting. It is essential to note, however, that these results cannot be generalized to environments in which anatomy is interpreted under different conditions, including differences in imaging protocols, levels of expertise, or methods of documenting anatomy.

### 3.9. Temporal Evolution of Microsurgical Practice

Clinical outcomes for patients undergoing microsurgery, as well as images acquired by imaging modalities, intraoperative assessment of anatomical integrity, and postoperative evaluation and follow-up of patients, have all undergone significant changes over the approximately 30 years captured within this registry.

The percentage of complete occlusions of treated aneurysms increased significantly from nearly 89% of patients during the period 1997–2007, to almost 93% during 2008–2014, and to almost 96% during 2015–2020 and 2021–2026 (*p*-trend < 0.001). There was a corresponding decrease in the percentage of branch-preservation failures from nearly 7% to less than 5% and then to slightly more than 2% across the respective time periods (*p* = 0.003).

There was little change in the percentage of high-complexity cases treated with microsurgical techniques, at approximately 17% across each of the four consecutive periods (*p* = 0.72). Thus, there were improvements in several technical aspects of microsurgery that did not appear to result from a decrease in the proportion of cases meeting the center’s criteria for a “high-complexity” case.

**Figure 5:**
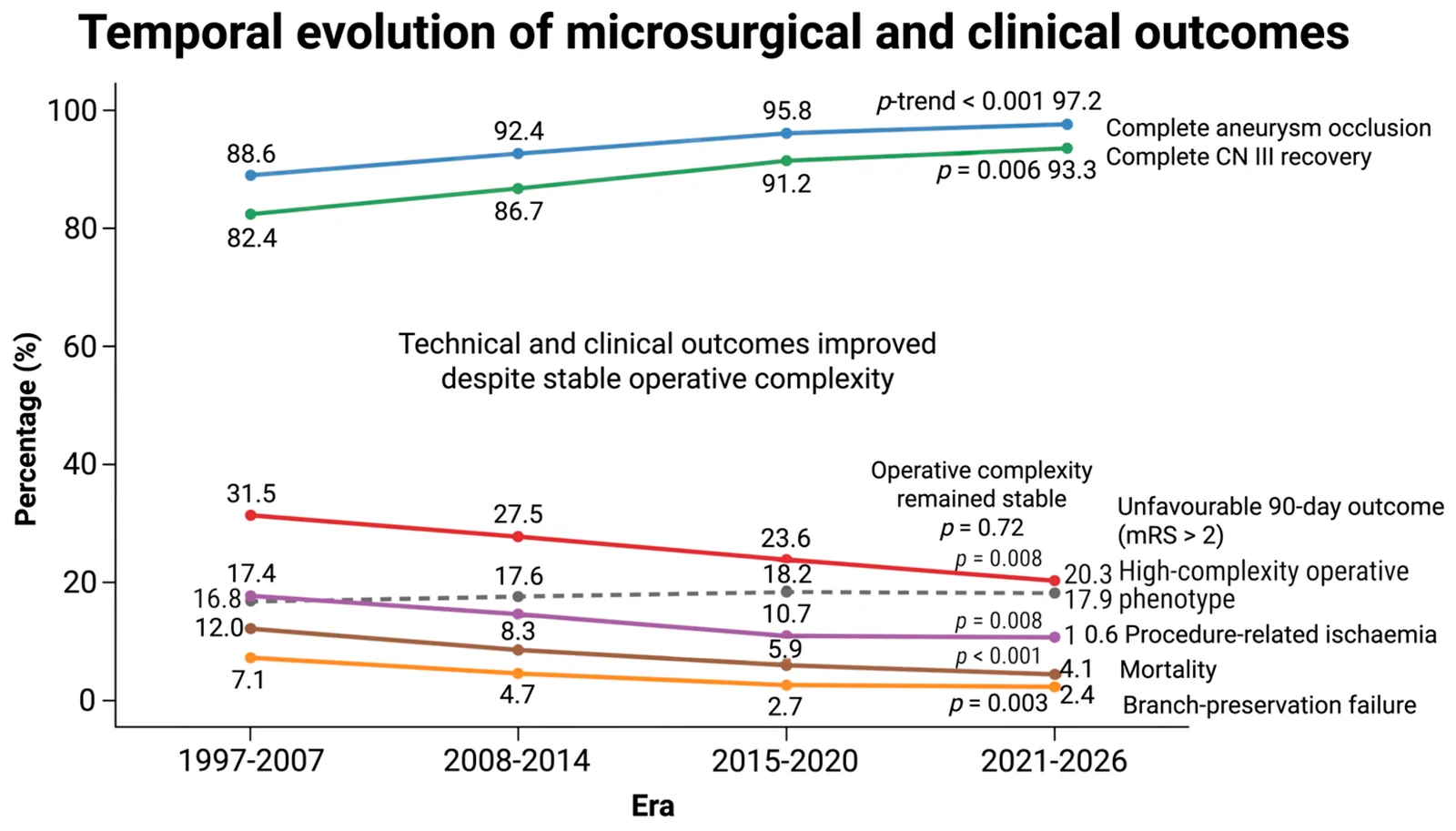
Temporal evolution of microsurgical and clinical outcomes in PComA aneurysm surgery. Complete aneurysm occlusion and complete CN III recovery increased across successive treatment eras, whereas unfavorable 90-day functional outcome (mRS > 2), procedure-related ischemia, mortality, and branch-preservation failure progressively declined.

Across the four different time periods, there was a corresponding decrease in intraoperative rupture rates from nearly 16% to approximately 9% (*p* = 0.014) and a marked increase in temporary clipping use from nearly 49% to just under 65% (*p* < 0.001). Additionally, utilization of intraoperative verification increased substantially for each modality, including ICG from approximately 13% to greater than 94%, micro-Doppler from approximately 19% to just under 87%, and intraoperative DSA from approximately 6% to greater than 41% (each *p* < 0.001). There were decreases in mortality rates from nearly 12% to approximately 4% and increases in complete recovery of CN III function from nearly 83% to greater than 93%. The temporal trends in microsurgical and clinical outcomes for patients undergoing microsurgical repair of PComA aneurysms are illustrated in Figure 5.

Temporal trends should not be interpreted causally. Due to concurrent changes in vascular imaging modalities, operative techniques, verification practices, postoperative ascertainment, perioperative care, and clip technologies, it is inappropriate to interpret these temporal trends as causal. Furthermore, these simultaneous changes may also affect interpretation of chronologically evaluated model performance. In particular, while same-center temporal analysis evaluates whether model performance remains robust when assessed in a later treatment era, it does not represent external validation. However, despite these limitations, PComA-C maintained good discriminative capability in the contemporary cohort. Nonetheless, center-defined complexity endpoints, together with the evolution of local surgical practices, may have contributed to that performance.

### 3.10. AI-to-Score Distillation and the PComA-CORE Clinical Instruments

The temporal AUC for the PComA-C score was 0.844, while the AUC for the full CatBoost model was 0.943. The PComA-C bedside score is a 0–19-point score derived by summing the following: neck width ≥ 4.5 mm = 4 points; dome-to-neck ratio ≤ 1.91 = 3 points; PComA sac adherence = 3 points; origin perforator = 3 points; parent ICA curvature ≥ 49.7° = 3 points; and PComA neck incorporation = 3 points. In the development data, the high-complexity phenotype occurred in 4/201 patients (2.0%) with low scores, 39/283 (13.8%) with intermediate scores, and 57/80 (71.3%) with high scores. In the same-center temporal cohort, the high-complexity phenotype occurred in 2/58 patients (3.4%) with low scores, 7/48 (14.6%) with intermediate scores, and 13/17 (76.5%) with high scores.

The PComA-R score is also based on six variables, each assigned a value between 0 and 3: aspect ratio ≤ 2.08 = 2 points; neck width ≥ 2.3 mm = 2 points; PComA neck incorporation = 2 points; P1 diameter ≤ 2.08 mm = 2 points; PComA sac adherence = 2 points; and perforator caliber ≥ 1.2 mm = 3 points, which can be summed to create a total score ranging from 0 to 13 points. Due to inconsistent trends of increasing risk across the lower levels of the initial three-level classification system, the score was retained as a continuous variable, providing limited exploratory utility as a potential indicator of higher risk. For purposes of exploratory assessment of predictive capability, scores ≥ 11 points were used to define “high” risk. Ischemia occurred in 30.4% (7/23) of patients with scores ≥ 11 compared with 11.0% (11/100) in those with scores < 11 points. The temporal AUC for the sparse PComA-R score was 0.644, while the AUC for the full model was 0.703. Thus, these results do not provide sufficient evidence to support the use of the sparse PComA-R score as a stand-alone clinical decision-making tool.

PComA-O was reduced to a 0–22-point derivation-stage score using the following six variables: duration of palsy ≥ 68.8 days = 6 points; CN III grooving = 5 points; complete palsy = 4 points; neck width ≥ 6.3 mm = 3 points; age ≥ 58.2 years = 2 points; and PComA diameter ≤ 1.15 mm = 2 points. The derivation-stage AUC was 0.968 (bootstrap 95% CI, 0.934–0.991). However, because all predictors and their respective thresholds were determined using the same development dataset, this estimate may be overly optimistic and should be interpreted accordingly. There were only two non-recovery events during follow-up of the most recent cohort, precluding meaningful temporal validation. Therefore, the thresholds established for the PComA-O score represent derivation-stage reference values rather than validated biological cutoffs.

The PComA-E score utilizes a combination of six variables to calculate a total risk score, with each variable scored as follows: ruptured presentation = 6 points; age ≥ 59.7 years = 4 points; NIHSS ≥ 4 = 4 points; baseline modified Rankin Scale (mRS) ≥ 1 = 3 points; admission Glasgow Coma Scale (GCS) ≤ 11 = 3 points; and AChA distance ≤ 3.11 mm = 2 points. Each variable contributes to a total possible score ranging from 0 to 22 points. Using this scoring system, development data showed that adverse 90-day outcomes occurred in 7.0% (9/128) of patients with low scores (0–5 points), 27.8% (55/198) of patients with intermediate scores (6–10 points), and 42.2% (81/192) of patients with high scores (≥11 points). In the same-center temporal cohort, adverse 90-day outcomes occurred in 4.3% (2/47), 15.4% (8/52), and 66.7% (12/18) of patients in the low-, intermediate-, and high-score groups, respectively. The temporal AUC for the sparse PComA-E score was 0.838, while the AUC for the full elastic-net model was 0.878.

As an illustration of the process whereby fully developed computational models can be distilled into more clinically accessible tools, important features of the candidate PComA-CORE scores—specifically retained variables, score architecture, identified risk gradients within the datasets, and same-center temporal evaluation where feasible—are listed in Table 12. These scores should be regarded as derivation-stage clinical anchors rather than externally validated biological cutoffs or treatment thresholds.

### 3.11. Integrated Behavior of the PComA-CORE Framework

To understand the integrated behavior of the PComA-CORE framework’s four components, it is essential to first evaluate them individually. Each component demonstrated predictive characteristics that suggest they function independently. This suggests treating them as individual elements rather than as one general “risk” model.

PComA-C demonstrated substantial dependency upon local anatomy. Temporal discrimination improved from 0.712 using clinical data alone to 0.804 by adding conventional morphological details and to 0.943 by incorporating specific details about PComA branch, perforator, neural, and geometric anatomy. However, the very high temporal AUC should be viewed cautiously since microsurgical complexity was evaluated at the originating center and potentially depends not solely on the anatomy of the PComA region but also on factors including the experience of the operating surgeon, the surgeon’s operative strategy, the hospital’s treatment protocols, and the treatment period. Thus, PComA-C’s performance does not provide evidence of reproducibility outside of the originating institution.

In contrast to PComA-C, PComA-R displayed distinctly different behavior. While PComA-R added discriminative capability through the use of perforator and vascular-domain information beyond that provided by traditional morphology, temporal discrimination was relatively modest (temporal AUC = 0.703), PPV was low (19.5%), and calibration was also imperfect. It appears reasonable to interpret PComA-R as being more representative of relative neurovascular susceptibility rather than as a clinically validated predictor of procedure-induced ischemia. As a result of its development from only 87 ischemic events, there remains considerable potential for residual overfitting as an explanation for the observed performance.

In contrast to PComA-C and PComA-R, PComA-O examined two non-equivalent aspects of CN III injury: duration of neurological impairment and degree of structural nerve damage. Duration of palsy, presence of grooving, and significant compression correlated with persistent deficits; nevertheless, only two instances of failure to recover occurred in the contemporaneous cohort. As a result, PComA-O represents an exploratory derivation-stage component without meaningful temporal validation or external validation.

Finally, PComA-E presented an alternative perspective regarding higher-dimensional modeling. Through penalized regression, baseline neurological severity alone resulted in a temporal AUC of 0.856. When all additional variables were added to baseline neurological severity, the resulting temporal AUC was only 0.878. These findings demonstrate that penalized regression performed as effectively as or better than many other more complex architectures, indicating that the analytical framework did not inherently favor greater computational complexity when greater complexity yielded minimal incremental information.

When viewed collectively, the primary contribution of PComA-CORE was not the prediction of four isolated outcomes but rather the integration of comprehensive neurovascular anatomy with four distinct biological and clinical outcomes: microsurgical complexity, susceptibility to procedure-related neurovascular injury, likelihood and progression of CN III recovery, and global functional status at 90 days. Moreover, the analyses permitted quantification of the incremental contribution of anatomical information to each outcome separately and indicated that anatomically detailed information yielded substantially different levels of incremental information for different types of questions, most notably microsurgical complexity versus global disability.

There are numerous constraints that limit interpretation of these results. For example, this study was retrospective and conducted at a single institution. Evaluation of the chronologically separated cohort remained within the confines of the same institution, with overlapping surgical expertise, measurement practices, and documentation systems. Therefore, true external validation has not been accomplished. Additionally, this registry spans nearly three decades, during which imaging quality, microsurgical techniques, clip technologies, intraoperative verification, postoperative imaging, and perioperative management have evolved significantly. The same-center temporal-split evaluations assessed performance during part of this evolutionary process but do not account for center- and era-specific differences in practice.

Additionally, the cohort analyzed contained only those aneurysms selected for microsurgical clipping. The results obtained cannot assess treatment-selection bias, cannot compare microsurgical clipping with contemporary endovascular treatments, and should not be utilized as an instrument for comparing clipping versus endovascular treatment. Finally, due to the availability of a large amount of anatomical detail at this high-volume microsurgical referral center, the level of detail documented in this registry likely exceeds what is commonly available in other hospitals.

Although the extent of phenotypic characterization possible through this registry may serve as a desirable reference point for structuring standardized assessments of neurovascular phenotypes in future studies, the feasibility and reproducibility of this approach across multiple centers remain uncertain.

Therefore, it appears that the value of this 779-variable registry arises more from its capacity to differentiate anatomically distinct domains and preserve temporal eligibility than from simply having a large number of available variables. The capacity to characterize incremental amounts of information represented by each domain, explore nonlinear relationships, estimate predictive uncertainty, and identify the amount of information that could potentially be incorporated into simplified candidate bedside scores also appears to contribute to the value of this registry.

Thus, PComA-CORE should be treated as a derivation framework with internal evaluation and same-center temporal evaluation, rather than as an externally validated clinical decision-making tool. The four components provide estimates of (1) anticipated microsurgical complexity; (2) relative susceptibility of the branch–perforator network to procedure-related injury; (3) probability and trajectory of CN III recovery; and (4) expected 90-day global functional status. These components are not designed to produce automated treatment decisions or replace expert clinical judgment.

These results provide support for continued exploration of anatomy-informed modeling in PComA aneurysm surgery; however, independent multicenter prospective validation will be required before either PComA-CORE or its derived bedside scores can be considered for routine clinical practice.

## 4. Discussion

### 4.1. Primary Finding

The primary finding of this study is that the four components of PComA-CORE—microsurgical complexity, neurovascular injury, oculomotor recovery, and overall functional outcome—are likely to represent fundamentally different prediction problems occurring within the same general anatomical region. Each of the four components of PComA-CORE arises within the PComA neurovascular environment, but they appear to require substantially different amounts and types of information for accurate prediction. This difference is especially important in PComA aneurysms because arterial geometry, collateral circulation, perforator topology, CN III location, and operative access are all situated within a highly confined anatomical space. While traditional concepts such as aneurysm size and neck morphology remain clinically relevant, they do not completely describe these relationships [12]. Therefore, it may not be feasible to define a single concept of “aneurysm risk” that adequately describes multiple clinically distinct processes. PComA-C was heavily dependent upon fine-scale anatomy; PComA-R suggested that distributed vascular susceptibility contributed to the process; PComA-O described relationships between the duration of neurological dysfunction and structural deformation of CN III; and PComA-E primarily reflected baseline neurological severity. It is also evident that dimensionality reduction may retain clinically relevant information for certain endpoints while eliminating important relationships for others [13].

Therefore, greater model complexity should not be viewed as an end goal in itself. Nonlinear modeling was beneficial when meaningful additional information was associated with detailed anatomical relationships, whereas a simpler penalized model was sufficient when most predictive information could be obtained from established clinical measures. As such, PComA-CORE not only provides a methodology for extracting additional signal but also provides a means of determining when increased computational complexity adds little benefit [14]. Traditionally, anatomical characterization of aneurysms has been based on quantifiable aspects such as size, neck morphology, rupture status, projection, occlusion, neurological complications, and functional outcome. These metrics remain important but do not fully explain how anatomically similar PComA aneurysms may represent substantially different microsurgical scenarios [15]. Factors such as PComA incorporation, P1 adequacy, AChA/perforator relationships, CN III deformation, and spatial constraints within the surgical corridor may contribute substantially to variability in operative difficulty without necessarily resulting in corresponding differences in postoperative outcomes. One of the key contributions of PComA-CORE is therefore its ability to distinguish between mechanisms and consequences rather than compressing multiple clinically distinct questions into a single estimator.

### 4.2. Different Predictive Behavior of the PComA-CORE Components

PComA-C indicates that microsurgical complexity is partly a spatial and reconstructive phenomenon. The combination of parent-vessel geometry, PComA incorporation, P1 configuration, perforator topography, and the relationships among the ICA, CN III, and skull-base corridor may ultimately determine operative difficulty. However, microsurgical complexity remains distinct from complications arising after successful completion of an operation. An operation that is technically challenging but ultimately successful remains anatomically complex.

However, given that PComA-C achieved very high same-center temporal performance, caution is required in interpreting this finding. Microsurgical complexity was defined at the originating center and is therefore likely to incorporate factors such as surgeon experience, operative strategy, institutional practice, and treatment era in addition to anatomy. Its performance therefore cannot be taken as evidence of external reproducibility. Additionally, converting the full model into a sparse bedside scoring system resulted in a loss of discrimination, illustrating the information lost when continuous variables and complex interactions are converted into discrete thresholds and additive points [16]. Thus, maintaining both a computationally rich model and a simpler version that sacrifices some information for improved interpretability and portability may be justified [17].

PComA-R demonstrates a different predictive paradigm. Ischemia secondary to clipping is unlikely to occur through a single anatomical pathway; instead, it may result from interactions among pre-existing vascular susceptibility, temporary occlusion, small-perforator injury, clip-induced distortion, thrombosis, hemodynamic effects, and other perioperative events. Although adding anatomical information related to perforators and vascular relationships improved predictive capability, discrimination remained modest (AUC = 0.703), PPV was low (19.5%), and calibration was imperfect. Given that only 87 ischemic events were available, residual overfitting also remains possible. Therefore, PComA-R should be viewed as an exploratory representation of relative neurovascular susceptibility rather than as a predictive instrument for individual procedure-related ischemic events.

PComA-O presents yet another distinct biological problem. Recovery of oculomotor function typically occurs over time and appears to depend upon both the duration of neurological dysfunction and the extent of structural deformation of CN III. Contact between an aneurysm and CN III provides less information than grooving or severe compression, while prolonged palsy may indicate more extensive neural injury or decreased reversibility. Therefore, survival-analysis methodologies may provide a more appropriate representation of recovery than classification at a single arbitrary postoperative time point [18]. Nonetheless, because only two non-recovery events occurred in the contemporary cohort, meaningful standalone temporal validation of PComA-O could not be performed. PComA-O should therefore remain an exploratory derivation-stage component, and its phenotypic associations should be viewed as hypothesis-generating until confirmed in larger cohorts with standardized neuro-ophthalmological follow-up [18]. PComA-E offers an alternative perspective compared with the anatomy-intensive components. Global disability is influenced by factors extending beyond the localized geometry of the PComA complex, including severity of the initial neurological deficit, age, premorbid functional status, systemic complications, perioperative cerebral injury, and neurological recovery. Baseline neurological status accounted for most of the predictive information; thus, detailed anatomy provided relatively limited additional discriminative value. The observation that penalized regression performed as well as or better than several more complex architectures supports the principle that high-dimensional modeling should be determined by the clinical question rather than by a preference for computational complexity.

### 4.3. Uncertainty and Intraoperative Verification

High-stakes prediction involves more than discrimination alone. Calibration, interpretability, and explicit representation of uncertainty are especially important when uncommon anatomical configurations are poorly represented. The uncertainty-aware analyses utilized here permitted ambiguous predictions rather than forcing assignment to a specific category. Therefore, although these findings do not constitute a formal demonstration of out-of-distribution detection, they support continued exploration of uncertainty as a potentially informative model output capable of identifying situations in which greater expert scrutiny may be warranted.

Intraoperative verification also serves as an intermediate state between preoperative anatomy and postoperative tissue injury. ICG, micro-Doppler, DSA, endoscopic visualization, and neuromonitoring can identify changes in vascular anatomy and flow that are unavailable before surgery [19,20]. Identification of abnormalities, corrective interventions, restoration of macroscopic flow, and ultimate tissue preservation represent related but distinct phenomena [21]. This distinction is particularly relevant for PComA-R because apparent preservation of macroscopic flow does not guarantee adequate perfusion of every small or incompletely visualized perforator territory.

Due to the retrospective nature of this study, no causal inference can be made that intraoperative verification or rescue prevented infarction. Instead, these observations support consideration of intraoperative verification as an intermediary phenotype between preoperative anatomical vulnerability and postoperative tissue-level injury.

### 4.4. Temporal Stability, Replicability and Clinical Relevance

Given that the registry spans nearly three decades, numerous advances in imaging modalities, clip technologies, microsurgical techniques, intraoperative verification, postoperative imaging, and perioperative care likely influenced both outcomes and their ascertainment [22]. The probability of detecting small postoperative ischemic lesions may therefore also have changed over time.

The proportion of high-complexity cases remained relatively stable over time, suggesting that improvements in outcomes were not solely attributable to progressive selection of anatomically easier cases. Some predictive relationships were preserved in the later cohort; however, this represents same-center temporal stability rather than evidence of transportability between centers [23]. Inter-observer agreement for major vascular, perforator, CN III, and quantitative anatomical measurements suggests that reproducibility of high-resolution anatomical variables is achievable within the present study environment [24]. Other centers using different image-acquisition protocols, reconstruction techniques, measurement standards, anatomical terminology, or levels of expertise may not achieve similar reproducibility [25]. Therefore, reproducibility should be evaluated rather than assumed during external validation.

Currently, PComA-CORE should be viewed as an anatomy-informed framework for structured clinical reasoning rather than as a clinical decision tool. The four components address complementary questions regarding anticipated microsurgical complexity, relative neurovascular susceptibility, CN III recovery, and global functional outcome. These estimates do not necessarily need to be concordant within an individual patient, which is one reason they were maintained as separate components. None should independently dictate treatment strategy or replace expert clinical judgment.

### 4.5. Limitations

Several limitations merit attention. First, this study was conducted retrospectively at a single center. Both cohorts remained within the same institution, with overlapping surgical expertise, imaging practices, measurement methods, and documentation systems. Thus, while this study provides internal evaluation and same-center temporal evaluation, it does not constitute external validation.

Second, this study spans nearly three decades. Advances in imaging sensitivity, microsurgical techniques, clip technologies, intraoperative verification, postoperative imaging, and perioperative care may have influenced both outcomes and their ascertainment. This is particularly relevant to the detection of small postoperative ischemic lesions.

Third, only aneurysms selected for microsurgical clipping were included in this cohort. Therefore, treatment-selection bias cannot be excluded and the current models cannot compare microsurgical clipping with contemporary endovascular strategies or determine the optimal treatment modality.

Fourth, some high-resolution anatomical features require expert interpretation. Although reproducibility was good within the present study, the level of anatomical detail available at this high-volume center may exceed that routinely available in other institutions. Standardization of definitions and imaging protocols, together with multicenter inter-observer studies, will therefore be required.

Finally, statistical support varied among components. There were too few contemporary non-recovery events for meaningful temporal validation of PComA-O. PComA-R had modest discrimination (AUC = 0.703), low PPV (19.5%), imperfect calibration, and only 87 ischemic events, leaving residual overfitting as a possibility. These components should therefore be interpreted according to their respective levels of validation and should not be considered evidence of causal relationships.

### 4.6. Future Validation

The next steps should involve prospective evaluation at independent centers. Multicenter studies should harmonize definitions relating to PComA/P1 configuration, branch incorporation, perforator topography, CN III deformation, and operative complexity, while imaging assessment and inter-observer reliability should be evaluated independently across participating institutions [26]. Both the full anatomy-informed models and their respective simplified bedside versions should be examined to determine whether gains in interpretability and portability justify reductions in discrimination. Standardized postoperative DWI and adjudication of lesion mechanism will be important for evaluating PComA-R. Larger cohorts containing sufficient numbers of persistent deficits, together with serial neuro-ophthalmological assessments, will be required for evaluating PComA-O. External validation will similarly be required to determine whether the simpler architecture of PComA-E continues to perform well in other populations [27]. Prospective evaluation of uncertainty-aware prediction will also be necessary to determine whether predictive uncertainty identifies cases in which additional expert evaluation modifies anatomical interpretation or subsequent clinical planning [28]. AI-to-score distillation should therefore be considered a translational rather than purely algorithmic challenge, with the objective of retaining clinically meaningful information while minimizing artificial precision.

Therefore, until these steps have been completed, PComA-CORE should continue to be considered a derivation framework with internal and same-center temporal evaluation, rather than an externally validated clinical decision-making tool.

## 5. Conclusions

The microsurgical approach to posterior communicating artery (PComA) aneurysms is more complex than one might initially predict. Microsurgical complexity, oculomotor recovery, neurovascular vulnerability, and the likelihood of achieving a favorable functional outcome all represent separate anatomically related but biologically distinct processes. We used the PComA-CORE framework as a way to disentangle each of these complexities in order to preserve the structure of clinical decision-making, rather than collapsing the entire treatment process into a single “risk” score. Each of the four components of PComA-CORE (C, O, R, and E) appeared to exist within a different predictive space. Operative complexity was highly dependent on fine-scale anatomical relationships between structures; neurovascular injury appeared to reflect a more generalized susceptibility to vascular injury across the PComA–P1–PCA–AChA–perforator network; recovery of CN III was primarily influenced by both the amount of time that nerve function had been compromised and the degree of structural deformation of the nerve itself; and overall functional outcome continued to be predominantly determined by preoperative clinical neurological status.

In a more general sense, our results indicate that there are many possible ways in which high-dimensional surgical data can be organized and presented, and that their potential utility does not necessarily reside in the sheer volume of information collected. Instead, we found that how variables were organized anatomically and temporally, and how they related to particular clinical questions, was considerably more important. Additionally, we found that increased computational complexity provided meaningful additional information when describing nonlinear anatomical interaction problems but added relatively little new predictive capability when most of the relevant information had already been captured through standard clinical measurements. Furthermore, reducing the fully parameterized models to their sparsest forms demonstrated that simplification is not information-neutral: some endpoints maintained clinically meaningful separation even after compression, whereas other endpoints appeared to rely upon complex relationships among multiple factors and therefore lost predictive information once compressed. Therefore, our goal in developing PComA-CORE was not simply to develop the best predictive model, but instead to identify what types of information are meaningful, when they become available, when they can be interpreted with reasonable reliability, and finally, how much of that information can be transformed into practical and clinically accessible forms.

We did not intend for PComA-CORE to serve as an automated system for making decisions about surgery or as a replacement for the individual cerebrovascular surgeon’s judgement. Our goal was to evaluate whether aspects of expert microsurgical thinking—anatomical difficulty, vascular vulnerability, potentially reversible neurological dysfunction, and expected neurological recovery—could be made more explicit, reproducible, and quantifiable. The observation that certain components of PComA-CORE remained informative over time, that high-resolution anatomical phenotypes were reproducible between observers, that predictive uncertainty could be represented, and that clinically relevant information was preserved after model distillation provides a basis for continuing to explore this work. However, based on our current analyses, we cannot recommend that PComA-CORE be considered ready for routine clinical use. Multicenter and prospective validation will therefore be necessary before determining whether these relationships remain reproducible in other settings. If such findings can be replicated across different institutions, observers, imaging environments, and surgical practices, PComA-CORE may provide a foundation for a broader form of human-supervised microsurgical intelligence in which computational models augment human reasoning by making expert thought more transparent, allowing clinicians to document their reasoning for educational purposes, improving communication of complex concepts to trainees and colleagues, and facilitating the transfer of microsurgical experience from one institution and generation of surgeons to another.

## Figures and Tables

**Table 1:** Core clinical and anatomical phenotype of the 687-patient PComA aneurysm cohort. The table provides a concise overview of the population from which the PComA-CORE instruments were derived, emphasizing variables that define the principal clinical and neurovascular phenotypes represented in the registry. Continuous variables are reported as median and interquartile range (IQR), and categorical variables as counts and percentages. CN III phenotype percentages are calculated only among patients presenting with preoperative oculomotor palsy. ICA, internal carotid artery; PCA, posterior cerebral artery; PComA, posterior communicating artery; CN III, oculomotor nerve. * Percentages calculated among the 175 patients with preoperative CN III palsy.

Variable	Category/Value	*n*	%
Cohort	Total	687	100.0
Age	Median, IQR	59.1 years	50.4–68.4
Sex	Female	531	77.3
	Male	156	22.7
Presentation	Ruptured	332	48.3
	Unruptured	355	51.7
Anatomical origin	ICA–PComA junction	653	95.1
	True proximal PComA	25	3.6
	True mid/distal PComA	9	1.3
Posterior circulation	Adult-type PComA	533	77.6
	Partial fetal PCA	88	12.8
	Complete fetal PCA	66	9.6
Preoperative CN III palsy	Present	175	25.5
CN III phenotype *	Partial	99	56.6
	Complete	76	43.4
Maximum aneurysm diameter	Median, IQR	6.2 mm	4.5–8.9
Neck width	Median, IQR	2.8 mm	1.7–4.4

**Table 2:** Principal information domains represented within the high-resolution longitudinal PComA registry. The registry architecture links baseline neurological condition with aneurysm morphology, posterior-circulation dependency, AChA and perforator anatomy, CN III relationships, operative access and reconstruction, intraoperative verification, and longitudinal recovery. The domains were used to structure feature eligibility and mechanistic interpretation rather than as predetermined statistical predictors. AChA, anterior choroidal artery; CN III, oculomotor nerve; GCS, Glasgow Coma Scale; mRS, modified Rankin Scale; PCA, posterior cerebral artery; PComA, posterior communicating artery; QoL, quality of life; SAH, subarachnoid hemorrhage; WFNS, World Federation of Neurosurgical Societies.

Domain	Representative Information	Principal Analytical Relevance
Clinical/SAH	Age, premorbid mRS, GCS, WFNS, Hunt–Hess, blood burden, hydrocephalus	Baseline severity and functional prediction
Aneurysm morphology	Size, neck, ratios, projection, irregularity, thrombus, calcification	Complexity and outcome
PComA–P1–PCA	Fetal configuration, P1 adequacy, diameters, collateral dependency	Posterior-circulation vulnerability
AChA	Distance, incorporation, adherence, branching pattern	Branch-preservation risk
Perforators	Number, origin, incorporation, concealment, premammillary/hypothalamic anatomy	Ischemic-risk modeling
CN III	Palsy phenotype, compression, grooving, stretching, distance, contact	Oculomotor-recovery modeling
Surgical access	Corridors, clinoidectomy, proximal/distal control, skull-base extensions	Complexity phenotype
Reconstruction	Clip number, geometry, reconstruction, repositioning	Operative burden
Verification/rescue	ICG, Doppler, DSA, endoscopy, neuromonitoring, flow rescue	Mechanistic and technical assessment
Longitudinal outcomes	mRS, GOS-E, CN III recovery, durability, cognition, QoL	Functional and recovery endpoints

**Table 3:** Temporal architecture of the PComA registry and analytical eligibility of predictor information. The seven-layer structure separates baseline and preoperative information from surgical planning, intraoperative execution, postoperative findings, and longitudinal outcomes. This temporal gating governed predictor eligibility throughout model development and ensured that information generated after an endpoint could not be used retrospectively to predict that endpoint.

Layer	Information Available	Representative Variables	Principal Role
T0	Presentation/baseline	Age, premorbid mRS, rupture, GCS, WFNS, Hunt–Hess, SAH burden	Preoperative prediction
T1	Preoperative anatomy	Morphology, PComA/P1, fetal PCA, AChA, perforators, CN III, spatial geometry	Core PComA-CORE predictors
T2	Surgical planning	Planned approach, control strategy, clinoidectomy, verification plan	Planning-enhanced analyses
T3	Intraoperative execution	Exposure, reconstruction, temporary clipping, ICG, Doppler, rescue	Complexity phenotype/update modeling
T4	Early postoperative state	CT, DWI, infarction, patency, residual aneurysm	Technical/ischemic endpoints
T5	Discharge/early recovery	mRS, GOS-E, NIHSS, deficit, death	Early clinical outcomes
T6	Longitudinal follow-up	30-day–24-month mRS, CN III recovery, durability, cognition, QoL	Long-term outcomes

**Table 4:** Predictor-domain completeness, sources of unavailable information, and missing-data handling in the PComA registry.

Variable/Endpoint	Eligible Population, *n*	Available, *n* (%)	Missing/Unavailable, *n* (%)	Structurally Non-Applicable in Full Cohort, *n* (%)
Sex	687	687 (100.0)	0 (0.0)	—
Rupture status	687	687 (100.0)	0 (0.0)	—
Anatomical origin	687	687 (100.0)	0 (0.0)	—
Posterior-circulation configuration	687	687 (100.0)	0 (0.0)	—
Preoperative CN III palsy status	687	687 (100.0)	0 (0.0)	—
CN III partial/complete phenotype	175	175 (100.0)	0 (0.0)	512 (74.5)
Pupillary involvement in PComA-O	175	175 (100.0)	0 (0.0)	512 (74.5)
CN III grooving in PComA-O	175	175 (100.0)	0 (0.0)	512 (74.5)
Aneurysm–CN III contact in PComA-O	175	175 (100.0)	0 (0.0)	512 (74.5)
SAH-specific core variables	332	320 (96.4)	12 (3.6)	355 (51.7)
Maximum aneurysm diameter	687	681 (99.1)	6 (0.9)	—
Neck width	687	678 (98.7)	9 (1.3)	—
PComA/P1 quantitative anatomy	687	658 (95.8)	29 (4.2)	—
AChA quantitative anatomy	687	628 (91.4)	59 (8.6)	—
Perforator topology	687	604 (87.9)	83 (12.1)	—
Surgical-corridor geometry	687	590 (85.9)	97 (14.1)	—
PComA-C endpoint classification	687	687 (100.0)	0 (0.0)	—
PComA-R endpoint classification	687	687 (100.0)	0 (0.0)	—
Early aneurysm occlusion status	687	589 (85.7)	98 (14.3)	—
90-day mRS	687	635 (92.4)	52 (7.6)	—
12-month mRS	687	531 (77.3)	156 (22.7)	—
12-month CN III recovery classification	175	175 (100.0)	0 (0.0)	512 (74.5)
Long-term recurrence/regrowth follow-up	687	586 (85.3)	101 (14.7)	—

**Table 5:** Data completeness and structural non-applicability for principal clinical, anatomical, and outcome variables in the PComA registry.

Predictor Domain	Eligible Population	Core-Domain Completeness	Missing/Incomplete	Structural Non-Applicability	Principal Source of Unavailable Data	Missing-Data Handling
Clinical/demographic	687	682/687 (99.3%)	5/687 (0.7%)	Minimal	Historical clinical documentation	Training-only imputation when required
SAH/rupture severity	332 ruptured	320/332 (96.4%)	12/332 (3.6%)	355 unruptured	Rupture-specific eligibility and historical documentation	Structural N/A not imputed; eligible missing values handled within training partitions
Conventional aneurysm morphology	687	670/687 (97.5%)	17/687 (2.5%)	None	Historical imaging measurement availability	Training-only imputation
PComA–P1–PCA anatomy	687	653/687 (95.1%)	34/687 (4.9%)	None	Imaging resolution and historical documentation	Training-only imputation
AChA anatomy	687	628/687 (91.4%)	59/687 (8.6%)	None	Small-vessel visualization and imaging resolution	Training-only imputation
Perforator topology	687	604/687 (87.9%)	83/687 (12.1%)	None	Imaging resolution and operative documentation	Training-only imputation; missingness indicators where appropriate
CN III clinical phenotype	175	173/175 (98.9%)	2/175 (1.1%)	512 without preoperative CN III palsy	Historical neurological/ophthalmological documentation	Structural N/A separated from missingness
CN III anatomical phenotype	175	168/175 (96.0%)	7/175 (4.0%)	512 without preoperative CN III palsy	Detailed spatial measurements and operative documentation	Training-only imputation where eligible
Surgical-corridor geometry	687	590/687 (85.9%)	97/687 (14.1%)	None	Historical imaging reconstruction capability	Training-only imputation; era-sensitive missingness indicators
Intraoperative verification status	687	672/687 (97.8%)	15/687 (2.2%)	Modality-specific	Historical technology availability and clinical indication	Availability/not-performed status separated from biological negative findings
Early postoperative ischemia assessment	687	654/687 (95.2%)	33/687 (4.8%)	None	MRI contraindication and historical imaging practice	Outcomes not imputed
90-day functional outcome	687	635/687 (92.4%)	52/687 (7.6%)	None	Follow-up availability	Not imputed
Longitudinal functional outcome	687	531/687 (77.3%) at 12 months	156/687 (22.7%)	None	Follow-up duration and availability	Not imputed

**Table 6:** Endpoint availability and event support across the four PComA-CORE components. The four components were developed in different endpoint-eligible populations according to their respective clinical questions.

PComA-CORE Component	Overall Eligible Population, *n*	Endpoint Available, *n* (%)	Endpoint Events/Outcome Distribution	Endpoint Missing, *n* (%)	Eligibility/Analytical Restriction
PComA-C	687	687 (100.0%)	122 high-complexity cases (17.8%); 565 non-high-complexity cases (82.2%)	0 (0.0%)	None
PComA-R	687	687 (100.0%)	87 procedure-related ischemic events (12.7%); 600 without procedure-related ischemia (87.3%)	0 (0.0%)	None
PComA-O	175	175 (100.0%)	155 complete recoveries by 12 months (88.6%); 20 with persistent oculomotor dysfunction (11.4%)	0 (0.0%)	Restricted to patients with preoperative aneurysm-related CN III palsy
PComA-E	687	635 (92.4%)	167 mRS >2 (26.3%); 468 mRS ≤2 (73.7%)	52 (7.6%)	90-day analysis restricted to patients with documented 90-day mRS

**Table 7:** Operational architecture of the four PComA-CORE instruments. PComA-C, PComA-O, PComA-R, and PComA-E were designed to address microsurgical complexity, oculomotor recovery, neurovascular preservation risk, and functional outcome, respectively. Although developed within a shared analytical framework, each instrument retained a mechanism-specific endpoint and predictor set to avoid conflating technically, anatomically, and neurologically distinct dimensions of PcomA aneurysm treatment.

Instrument	Principal Clinical Question	Overall Endpoint-Eligible Population	Prediction Horizon	Dominant Information Domains
PComA-C	How technically complex is clipping likely to be?	687 patients; 122 high-complexity cases	Pre-incision	Morphology, corridors, fetal/P1 anatomy, AchA, perforators, CN III
PComA-O	Will CN III recover, and how rapidly?	175 preoperative palsies; time to complete recovery	Pre-incision	Palsy phenotype, duration, pupil, compression, grooving, contact geometry
PComA-R	How vulnerable are critical branches and perforators?	687 patients; 87 procedure-related ischemic events	Pre-incision	Fetal dependency, P1, PcomA/AchA incorporation, perforators
PComA-E	What 90-day functional outcome is expected?	635 with 90-day mRS; 167 mRS > 2	Pre-incision	Clinical severity plus PcomA-specific anatomy

**Table 8:** Multilayer statistical, machine-learning, and artificial-intelligence architecture used for PComA-CORE development. Model families were selected to provide complementary strengths in interpretability, regularization, nonlinear interaction modeling, longitudinal prediction, and high-performance tabular learning. Advanced architectures were considered benchmarks rather than inherently superior models and were retained only when their performance gains were supported by calibration, resampling stability, and temporal validation.

Model Family	Representative Methods	Principal Purpose
Interpretable regression	Logistic/Firth, ordinal regression, Cox, flexible survival	Reference modeling and effect estimation
Sparse/penalized models	Ridge, LASSO, elastic net	Collinearity control and stable reduction
Bagging	Random Forest, Extra Trees	Nonlinear interaction benchmark
Gradient boosting	XGBoost, LightGBM, CatBoost	High-performance tabular prediction
Glass-box AI	Explainable Boosting Machine	Interpretable nonlinear risk functions
Foundation/deep tabular AI	TabPFN; regularized tabular transformer	Contemporary AI benchmark
Survival ML	Random Survival Forest, boosted survival, XGBoost-AFT	CN III recovery trajectory
Ensemble intelligence	Cross-fitted stacking	Consensus prediction when demonstrably beneficial
Clinical distillation	Sparse integer optimization	Translation of AI signal into bedside scores

**Table 9:** Multidimensional framework for evaluation of the PComA-CORE prediction models and derived clinical scores. Performance assessment extended beyond conventional discrimination metrics to evaluate probability calibration, classification behavior, ordinal and survival prediction, decision-curve utility, explanation stability, uncertainty, temporal generalizability, anatomical-domain contribution, and the degree of predictive information retained after conversion of full AI models into sparse integer scores.

Domain	Principal Measures
Discrimination	ROC AUC, PR-AUC, C-index, time-dependent AUC
Classification	Sensitivity, specificity, PPV, NPV, balanced accuracy, F1, MCC
Calibration	Brier score, intercept, slope, integrated calibration error/index, calibration curves
Ordinal performance	Somers’ Dxy, ordinal concordance, weighted κ, ranked probability score
Survival performance	C-index, integrated Brier score, time-dependent discrimination and calibration
Clinical utility	Decision-curve net benefit
Interpretability	SHAP, ALE, EBM functions, interaction analysis, feature-rank stability
Uncertainty	Bootstrap variance, posterior uncertainty, conformal coverage, abstention
Transportability	Temporal validation, subgroup stress testing, treatment-era analysis
Anatomical contribution	Domain-ablation analysis
Clinical compression	Performance retained by sparse integer PComA-CORE scores

**Table 10:** High-resolution neurovascular and outcome phenotype of the PComA registry. The table emphasizes branch, perforator, cranial-nerve, technical, neurological, and longitudinal information complementary to the demographic characteristics presented in Table 1. AChA, anterior choroidal artery; CN III, oculomotor nerve; mRS, modified Rankin Scale; PCA, posterior cerebral artery; PComA, posterior communicating artery. * Among patients with available early occlusion assessment. † Among patients presenting with preoperative CN III palsy.

Domain	Endpoint	Result
PComA anatomy	Neck incorporation	143/687 (20.8%)
	Sac incorporation	51/687 (7.4%)
	Sac adherence	118/687 (17.2%)
AChA anatomy	Neck incorporation	57/687 (8.3%)
	Sac adherence	62/687 (9.0%)
Perforator anatomy	Neck incorporation	105/687 (15.3%)
	Hidden retro-dome perforators	127/687 (18.5%)
	Stretched over aneurysm dome	264/687 (38.4%)
CN III anatomy	Direct aneurysm contact	264/687 (38.4%)
	Grooving	77/687 (11.2%)
Operative phenotype	High-complexity procedure	122/687 (17.8%)
	Temporary clipping	384/687 (55.9%)
	Intraoperative rupture	85/687 (12.4%)
Technical outcome	Composite technical success	534/687 (77.7%)
	Complete early occlusion *	551/589 (93.5%)
Vascular preservation	PComA/PCA patent	667/687 (97.1%)
	AChA patent	676/687 (98.4%)
	Visualized perforators patent	663/687 (96.5%)
Ischemic outcome	Procedure-related ischemia	87/687 (12.7%)
	Silent imaging-only ischemia	53/87 (60.9%)
Functional outcome	90-day mRS 0–2	468/635 (73.7%)
	90-day mRS > 2	167/635 (26.3%)
CN III recovery †	Complete recovery by 90 days	112/175 (64.0%)
	Complete recovery by 12 months	155/175 (88.6%)
	Complete recovery by 24 months	156/175 (89.1%)
Durability	Recurrence/regrowth	13/687 (1.9%)
	Retreatment	14/687 (2.0%)

**Table 11:** Comparative discrimination of the principal statistical, machine-learning, interpretable-AI, foundation-model, and ensemble approaches. Internal estimates represent out-of-fold performance; temporal estimates were obtained from the chronologically isolated validation cohort. Models were selected according to discrimination, calibration, temporal stability, interpretability, and parsimony rather than AUC alone.

Model	PComA-C Internal/Temporal AUC	PComA-R Internal/Temporal AUC	PComA-O 12-Month AUC	PComA-E Internal/Temporal AUC
Logistic/Firth regression	0.849/0.858	0.632/0.648	—	—
Elastic net	0.873/0.886	0.651/0.662	0.861	0.883/0.878
Random Forest	0.889/0.907	0.664/0.681	0.858	0.872/0.861
Extra Trees	0.896/0.918	0.672/0.694	0.885	0.878/0.864
XGBoost	0.904/0.931	0.673/0.695	0.902	0.879/0.869
LightGBM	0.898/0.922	0.668/0.688	0.892	0.875/0.866
CatBoost	0.911/0.943	0.681/0.703	0.888	0.881/0.871
EBM	0.882/0.895	0.659/0.672	0.871	0.866/0.854
TabPFN	0.877/0.884	0.645/0.654	0.848	0.858/0.841
Tabular transformer	0.869/0.872	—	—	—
Stacked ensemble	0.918/0.932	0.684/0.698	—	—

**Table 12:** Candidate sparse PComA-CORE instruments derived from the complete computational architecture. Thresholds and integer weights were estimated within development data and should be interpreted as derivation-stage clinical anchors rather than externally validated biological cut-offs or treatment thresholds. PComA-R remained more informative as a continuous anatomical-risk representation, while PComA-O requires dedicated external validation because of sparse contemporary non-recovery events.

Instrument	Retained Preoperative Features	Score Architecture	Temporal Behavior	Performance
PComA-C	Neck width; dome/neck ratio; PComA adherence; P1-origin perforator; ICA curvature; PComA neck incorporation	0–19; low 0–4, intermediate 5–11, high 12–19	Complexity: 3.4% → 14.6% → 76.5%	Temporal AUC 0.844
PComA-O	Palsy duration; CN III grooving; complete palsy; neck width; age; PComA diameter	0–22; derivation-stage	Temporal categorical validation limited by only 2 non-recovery events	Derivation AUC 0.968 (95% CI, 0.934–0.991)
PComA-R	Aspect ratio; neck width; PComA incorporation; P1 diameter; PComA adherence; perforator caliber	0–13; retained primarily as continuous score	≥11 points: 30.4% ischemia vs. 11.0% below 11	Temporal AUC 0.644
PComA-E	Rupture; age; NIHSS; baseline mRS; admission GCS; AChA distance	0–22; low 0–5, intermediate 6–10, high ≥11	mRS > 2: 4.3% → 15.4% → 66.7%	Temporal AUC 0.838

## Data Availability

The data presented in this study are available from the corresponding author upon reasonable request. The data are not publicly available because they contain participant-level information subject to confidentiality, ethical, and data-protection requirements and unrestricted public dissemination was not covered by the applicable ethical and informed-consent framework. The code and modeling pipeline are publicly available at https://github.com/INNBN-data/PComA-CORE (accessed on 17 August 2026).

## References

[1] Jeong S.Y., Jung S.C., Roh Y.H., Kwon S.U., Kang D.-W., Kim J.S., Choi K.M., Kim S., Jeong E. (2025). Serial Changes and Optimal Imaging Windows in Vessel Wall MRI for Unruptured Intracranial Artery Dissection. Sci. Rep..

[2] Oprea S., Pantu C., Breazu A., Munteanu O., Dumitru A.V., Radoi M.P., Costea D., Baloiu A.I. (2026). A Ruptured Tri-Lobulated ICA–PCom Aneurysm Presenting with Preserved Neurological Function: Case Report and Clinical–Anatomical Analysis. Diagnostics.

[3] Toader C., Serban M., Covache-Busuioc R.-A., Radoi M.P., Aljboor G.S.R., Costin H.P., Ilie M.-M., Popa A.A., Gorgan R.M. (2025). Single-Stage Microsurgical Clipping of Multiple Intracranial Aneurysms in a Patient with Cerebral Atherosclerosis: A Case Report and Review of Surgical Management. J. Clin. Med..

[4] Attachaipanich T., Khawaja M., Takahashi E.A., Virk H.U.H., Alam M., Gilchrist I.C., Krittanawong C. (2026). Percutaneous Coronary Intervention and Vascular Access Complications: A Contemporary Review. npj Cardiovasc. Health.

[5] Saito S., Hasegawa H., Takino T., Ando K., Shibuya K., Takahashi H., On J., Suzuki T., Oishi M., Fujii Y. (2021). Unilateral Oculomotor Nerve Palsy Caused by Arterial Compression Accompanying Subarachnoid Hemorrhage: A Case Report. Acta Neurochir..

[6] Han Y.-F., Jiang P., Tian Z.-B., Chen X.-H., Liu J., Wu Z.-X., Gao B.-L., Ren C.-F. (2022). Risk Factors for Repeated Recurrence of Cerebral Aneurysms Treated with Endovascular Embolization. Front. Neurol..

[7] Chen X., Li H., Wang M.-Z., Li M., Cao Y., Zhang D., Zhang Y., Wang H., Wang S. (2020). Clinical Features and Outcomes of PComA Aneurysms Originating from Fetal Posterior Communicating Arteries in a Single Institution. Chin. Neurosurg. J..

[8] Tataru C.-I., Pantu C., Breazu A., Brehar F.-M., Serban M., Covache-Busuioc R.-A., Toader C., Munteanu O., Radoi M.P., Dumitru A.V. (2026). Branch-Critical Clipping of a Ruptured Carotid–Posterior Communicating Aneurysm with Fetal PCA Configuration. Diagnostics.

[9] Dreyer M., Berend J., Labarta T., Vielhaben J., Wiegand T., Lapuschkin S., Samek W. (2025). Mechanistic Understanding and Validation of Large AI Models with SemanticLens. Nat. Mach. Intell..

[10] Dueñas G., Jimenez S., Ferro G.E.M. (2026). Unveiling the Black Box of Item Difficulty: An Interpretable Decomposition Approach Using LLM-Based Option Plausibility. AI.

[11] Collins G.S., Moons K.G.M., Dhiman P., Riley R.D., Beam A.L., Calster B.V., Ghassemi M., Liu X., Reitsma J.B., van Smeden M. (2024). TRIPOD+AI Statement: Updated Guidance for Reporting Clinical Prediction Models That Use Regression or Machine Learning Methods. BMJ.

[12] Zhang J., Can A., Lai P.M.R., Mukundan S., Castro V.M., Dligach D., Finan S., Yu S., Gainer V.S., Shadick N.A. (2020). Age and Morphology of Posterior Communicating Artery Aneurysms. Sci. Rep..

[13] Chen B., Guo J., Gong C., Zhu C., Wu Y., Wang S., Zheng Y., Lu H. (2025). Proteomic Analysis of Spinal Dorsal Horn in Prior Exercise Protection against Neuropathic Pain. Sci. Rep..

[14] Tian T., Liu S., Ji G. (2026). Interpretable Machine Learning Unveils Non-Linear Inflammatory Thresholds and Synergistic Interactions in Post-Burn Hypertrophic Scarring: Development of an Intelligent Clinical Decision Support System. Sci. Rep..

[15] Duan Z., Li Y., Guan S., Ma C., Han Y., Ren X., Wei L., Li W., Lou J., Yang Z. (2018). Morphological Parameters and Anatomical Locations Associated with Rupture Status of Small Intracranial Aneurysms. Sci. Rep..

[16] Elnahla M., Guo Y., Atadero R.A. (2026). A New Gaussian Process Regression-Based Approach to Leverage Non-Destructive Evaluation Data in Bridge Deterioration Prediction Models. Eng. Struct..

[17] Bolou K., Triantafyllou G., Arkoudis N.-A., Papadopoulos-Manolarakis P., Chatziralli I., Papadopoulos V., Velonakis G., Piagkou M. (2026). Oculomotor Nerve Palsy—Etiologies, Symptoms and Diagnosis: A Systematic Review with Meta-Analysis. Diagnostics.

[18] Beydoun M.A., Noren Hooten N., Beydoun H.A., Georgescu M.F., Tsai J., Evans M.K., Zonderman A.B. (2026). Epigenetic Aging Markers in the Association between Frailty and Mortality among U.S. Adults. BMC Med..

[19] Khubzan W.D., Alharati S.A., Aljuaid R.W., Algethami D.S., Alotibi S.S., Alotaibi H.M., Aljuaid S.S., Almalki R.S., Mahfouz M.E.M. (2026). The Digital Evolution of Surgical Planning: A Systematic Review of Immersive and Interactive Technologies. Front. Surg..

[20] Vintimilla Rivadeneira V., Leon-Rojas J.E. (2025). Advances in Preoperative and Intraoperative Technologies for Safe Resection of Gliomas in Cognitive Regions. Cancers.

[21] Shickel B., Loftus T.J., Ruppert M., Upchurch G.R., Ozrazgat-Baslanti T., Rashidi P., Bihorac A. (2023). Dynamic Predictions of Postoperative Complications from Explainable, Uncertainty-Aware, and Multi-Task Deep Neural Networks. Sci. Rep..

[22] Guo G., Zhao L., Wu R., Xue B., Zhang S., Liang H., Gao T., Sun Y., Liu Y., Li C. (2022). Case Report: The Different Fates of Three Aneurysms: Diagnosis and Treatment Strategies for Unruptured Intracranial Aneurysms with Other Intracranial Diseases. Front. Surg..

[23] Liu X., Scherrer S., Egger S., Lauber B., Taube W., Xin L. (2026). Functional and Structural Connectome-Based Predictive Modelling of Balance in Elderly Adults. Sci. Rep..

[24] Quinn L., Tryposkiadis K., Deeks J., De Vet H.C.W., Mallett S., Mokkink L.B., Takwoingi Y., Taylor-Phillips S., Sitch A. (2023). Interobserver Variability Studies in Diagnostic Imaging: A Methodological Systematic Review. Br. J. Radiol..

[25] Sabanci K., Aslan B., Aslan M.F. (2026). Medical Image Segmentation Methods: A Decision-Guided Survey Covering 2D/3D CNNs, Transformers, VLMs, SAM-Based Models and Diffusion Approaches. Bioengineering.

[26] Csipo T., Lipecz A., Mukli P., Péterfi A., Szarvas Z., Ungvari A., Alaoui L.E., Sándor M., Kállai A., Fekete M. (2025). Advancing Prediction of Age-Related Vascular Cognitive Impairment Based on Peripheral and Retinal Vascular Health in a Pilot Study: A Novel Comprehensive Assessment Developed for a Prospective Workplace-Based Cohort (The Semmelweis Study). GeroScience.

[27] Li Z., Fan Y., Ma J., Wang K., Li D., Zhang J., Wu Z., Wang L., Tian K. (2024). The Novel Developed and Validated Multiparametric MRI-Based Fusion Radiomic and Clinicoradiomic Models Predict the Postoperative Progression of Primary Skull Base Chordoma. Sci. Rep..

[28] Riley R.D., Collins G.S., Kirton L., Snell K.I., Ensor J., Whittle R., Dhiman P., van Smeden M., Liu X., Alderman J. (2025). Uncertainty of Risk Estimates from Clinical Prediction Models: Rationale, Challenges, and Approaches. BMJ.

